# Metagenomic Assessment of Full-Scale Wastewater Treatment Plants Identifies Sentinel Antibiotic Resistance Gene Families for Monitoring Reclaimed Wastewater and Treated Sludge

**DOI:** 10.1021/acs.est.5c13541

**Published:** 2026-02-04

**Authors:** Liam P Brown, Anna Marizzi, Carles M Borrego, Giulia Gionchetta, Zhao Zhengzheng, Rodrigo B Carneiro, Pablo Gago-Ferrero, Victor Matamoros, Jessica Subirats

**Affiliations:** † Ottawa Laboratory (Carling), 5737 Canadian Food Inspection Agency, 960 Carling Avenue, bldg.22, Ottawa, Ontario K1A 0C6, Canada; ‡ Department of Environmental Chemistry, 203229 Institute of Environmental Assessment and Water Research (IDAEA), Severo Ochoa Excellence Center, Spanish Council of Scientific Research (CSIC), Jordi Girona 18-26, Barcelona E-08034, Spain; § Catalan Institute for Water Research (ICRA-CERCA), Emili Grahit 101, Girona E-17003, Spain; ∥ Grup d’Ecologia Microbiana Molecular, Institut d’Ecologia Aquàtica, 16738 Universitat de Girona, Campus de Montilivi, Girona E-17003, Spain; ⊥ National Key Laboratory of Agricultural microbiology, College of Life Science and Technology, National Engineering Research Center of Microbial Pesticides, 47895 Huazhong Agricultural University Wuhan 430070, PR China; # Laboratory of Chromatography (CROMA), 28133 São Carlos Institute of Chemistry, University of São Paulo (USP), 400, Trabalhador São-Carlense Ave., São Paulo, São Carlos 13566-590, Brazil

**Keywords:** reclaimed water, sludge, antibiotic resistance, wastewater treatment, metagenomics, monitoring

## Abstract

The new European (EU) regulation on water reuse explicitly incorporates antimicrobial resistance (AMR) into routine monitoring and risk management, creating an urgent need to define target antibiotic resistance genes (ARGs) for reclaimed irrigation water and agricultural sludge. However, existing global data largely focus on secondary effluents, providing little actionable evidence for reuse-oriented systems. Here, we present the first integrated framework combining targeted antibiotic residue analysis with shotgun metagenomics of the resistome, mobilome, and microbiome across full-scale reuse-oriented wastewater treatment plants (WWTPs) in Southern Europe to identify sentinel antibiotic resistance families for monitoring. Reclaimed effluents exhibited lower AMR exposure levels than those typically reported for secondary effluents (<0.5 ARGs/cell), while mobile genetic element (MGE) abundances were comparable to secondary effluents (1–2 MGEs/cell). Effluent communities differed by WWTP configuration: membrane bioreactor combined with ultrafiltration favored nutrient-removal/oxidative-stress taxa and reduced transferable MGEs, whereas plants relying on physical separation (sand filtration or reverse osmosis) retained fecal-associated taxa and MGEs. Specific clinically relevant ARGs persisted after treatments, including *aadA* and *aph­(3′’)-Ibs* (resistance to aminoglycosides), *ermB* and *mphA* (resistance to macrolides), and *bla*_OXA‑129_ (resistance to beta-lactams), which we identify as sentinel markers for monitoring reclaimed water and sludge. We advance a generalizable two-step framework, metagenomic discovery to identify sentinel markers, followed by targeted assays for streamlined surveillance, that provides the first operational blueprint for integrating AMR into water reuse management under the EU regulation.

## Graphical abstract

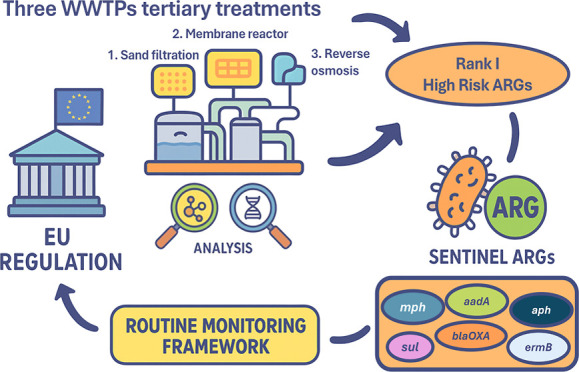

## 1 Introduction

Water scarcity is one of the most critical environmental and socio-economic challenges of the 21st century, aggravated by population growth, urbanization and climate change. According to the European Environment Agency, water scarcity affected, on average, 30% of European Union (EU) territory and 34% of its population over the past decade, with the Mediterranean region standing out as especially vulnerable due to its semiarid climate, high population density, and pronounced climatic variability.^1^

Under this scenario, reclaimed water offers a sustainable response to water scarcity. The potential for water reuse is particularly relevant for agriculture, which is the largest consumer of water globally and accounts for over 60% of total water use in many Mediterranean countries.^2–4^ Spain reuses approximately 400 hm^3^/year of treated wastewater, or about 12% of its total municipal wastewater production, with agricultural usage of reclaimed wastewater varying by region. The technical and economic potential for irrigation reuse is estimated to range between 1200 and 1300 hm^3^/year.^5^ Public acceptance of water reuse remains limited, largely due to concerns regarding potential risks to human, animal, and environmental health. In the EU, agricultural water reuse is governed by regulation (EU) 2020/741, which sets minimum requirements for water quality, monitoring, and risk management. For the first time, this regulation explicitly recommends the incorporation of antimicrobial resistance (AMR) into monitoring and risk management. To comply with these standards, reclaimed water must undergo advanced treatment processes beyond conventional wastewater treatment. These treatments include membrane filtration (ultra- and nanofiltration), reverse osmosis, ultraviolet (UV) disinfection, ozonation, and activated carbon adsorption, technologies designed to remove emerging contaminants, including trace organic pollutants and pathogenic microorganisms that are not fully eliminated by conventional secondary treatment.^6^ Although these advanced treatments significantly improve water quality, many studies have shown that reclaimed water can still contain antibiotic residues, antibiotic-resistant bacteria (ARB), and antibiotic resistance genes (ARGs).^7–9^ Irrigation with such water may contribute to the dissemination of AMR in soils and crops, and consequently the food chain.^10^ To manage this risk under the new regulatory framework, there is a need to identify sentinel ARGs, understood as clinically relevant resistance genes that are representative of high-risk determinants, and whose persistence and mobility make them suitable markers for monitoring AMR dissemination. Operationalizing the regulation therefore requires: (i) robust identification of sentinel ARG targets, (ii) understanding how treatment configurations influence their fate, and (iii) practical assays for routine surveillance. Public AMR surveillance platforms currently contain little to no data derived from WWTP effluents or reclaimed water systems (Van Boeckel et al., 2019; The Center for Disease Dynamics, 2021).^11,12^ In parallel, most studies profiling ARGs mainly focused on secondary effluents, with relatively few examining reclaimed water, and have often relied on targeted quantification of selected ARGs using qPCR or high-throughput quantitative PCR (HT-qPCR).^13–17^ Despite some studies have already applied shotgun metagenomics to characterize resistomes in reclaimed water, these investigations were largely descriptive and did not establish a framework for identifying sentinel ARGs that represent high-risk indicators suitable for routine monitoring in water reuse systems.^18,19^ As a result, the interpretability of existing AMR data for reuse-oriented management and regulatory applications remains limited.

In this study, we address this gap by coupling targeted antibiotic residue measurements with shotgun metagenomics to jointly characterize bacterial communities, ARGs, and mobile genetic elements (MGEs) in influent, reclaimed effluents, and anaerobically digested sludge from three full-scale WWTPs with distinct reuse-oriented tertiary treatments. Our objectives are to (i) determine how treatment configuration and antibiotic residues influence the fate of ARG target drug classes, high-risk ARGs, transferable MGEs, and human pathogens, and (ii) identify recalcitrant clinically relevant ARGs as sentinel markers for monitoring AMR in reclaimed water and sludge. We establish sentinel antibiotic resistance families as management-ready indicators and advance a generalizable framework, metagenomic discovery followed by targeted assays, to integrate AMR monitoring into water reuse practices.

## 2 Material and Methods

### 2.1 Sample Collection

Influent, effluent and sludge samples were collected from three reuse-oriented municipal wastewater treatment plants (WWTPs) in the metropolitan area of Barcelona (Spain), once per week over three consecutive weeks in February 2024 to obtain three biological replicates. Influent and effluent samples were collected as 24 h flow-proportional composites to ensure representative average conditions. Sludge samples of the same WWTPs were collected after anaerobic digestion treatment, dehydration, and drying. The sludge from these WWTPs is commonly applied as an organic fertilizer to agricultural soils used for cereal crops in Catalonia. The three WWTPs treat both sewage and pretreated industrial water and are configured for water reuse. The Prat de Llobregat WWTP (hereafter P.LL) has a maximum treatment capacity of 420,000 m^3^ per day, serving over 2 million residents and processing approximately 36% of the total wastewater from the Barcelona metropolitan area. The tertiary treatment at P.LL WWTP includes microfiltration, reverse osmosis, ultraviolet (UV) disinfection, and chlorination, resulting in reclaimed water for industrial applications, irrigation, and other uses. The Sant Feliu de Llobregat WWTP (hereafter F.LL) processes 72,000 m^3^ per day, serving an equivalent of 320,000 residents, which corresponds to approximately 7% of the total wastewater from the Barcelona metropolitan area. Its tertiary treatment includes sand filtration and UV disinfection and chlorination, producing reclaimed water suitable for agricultural irrigation. The Gavà-Viladecans WWTP (hereafter G-V) treats 64,000 m^3^ per day, serving an equivalent of 300,000 residents, representing 5% of the total wastewater from the Barcelona metropolitan area. The tertiary treatment at G-V includes membrane bioreactor (MBR) with ultrafiltration, UV disinfection, and chlorination yielding reclaimed water for agricultural irrigation and hydraulic restoration.

### 2.2 Analyses of Antibiotic Residues in Collected Samples

#### 2.2.1 Sample Preparation and Extraction

For water samples, 24 h flow-proportional composite samples (100 mL aliquots) were filtered through Whatmann GF/F glass microfiber filters and extracted following a procedure described elsewhere.^20^ In brief, samples were acidified to pH = 2–3 with HCl, and 100 μL of 0.1 M EDTA and 25 μL of an antibiotic Surrogate Mix (1 ppm) were added (Supporting Information Table S1). Samples were loaded onto preconditioned SPE cartridges (OSASIS HLB, 6 cm^3^ (200 mg) Extraction Cartridges) using vacuum. Cartridges were dried and stored at −80 °C until analysis. Before injection, cartridges were eluted with 6 mL of methanol (MeOH) (Merck), eluates were evaporated under a constant nitrogen stream, and residues were reconstituted with 500 μL of HPLC-grade water (Merck). Finally, samples were ultrasonicated for 5 min and filtered through a Micro-Spin centrifuge tube with 0.2 μm PTFE microfiltration membrane (Frisenette).

Sludge samples were collected from each WTTP and stored at −80 °C. Antibiotics were extracted following a procedure described elsewhere.^21^ Approximately 0.5 g of sludge was weighed and transferred into 50 mL Falcon tubes. Samples were subsequently spiked with 40 μL of Antibiotic Surrogate Mix at 1 ppm (Supporting Information Table S1) and left for 30 min at room temperature. For extraction, 4 mL of McIlivain buffer and 1 mL of acetonitrile were added to each sample. After ultrasonication for 15 min, 2 mL of lead acetate was added. Samples were vortexed for 1 min and centrifuged at 5000 rpm for 15 min. The supernatant was transferred into a new 20 mL glass vial, and 13 mL of 0.2 M EDTA were added. For the cleanup, Strata-X (200 mg/6 mL) solid phase extraction (SPE) cartridges were preconditioned with 5 mL of MeOH and 5 mL of HPLC-Water (Merck). Extracts were loaded onto the cartridges, rinsed with 1 mL of H_2_O/MeOH (95/5, v/v). Cartridges were subsequently dried and stored at −80 °C. Prior to injection, cartridges were eluted with 5 mL of MeOH, the eluates were evaporated under a constant nitrogen stream, and residues were reconstituted with 1 mL of HPLC-water. Samples were then ultrasonicated for 5 min and filtered through a Micro-Spin centrifuge tube with 0.2 μm PTFE microfiltration membrane (Frisenette).

#### 2.2.2 Detection and Quantification of Antibiotic Residues

A volume of 10 μL of water sludge sample extracts were injected into a UHPLC-qTOF instrument (Bruker-Impact II) with an Intensity Solo C18–2 column (100 × 2.1 mm, 1.8 μm) in electrospray ionization (ESI) positive mode. The water mobile phase consisted of 99% HPLC grade water, 1% MeOH and 5 mM ammonium acetate, while the organic mobile phase consisted of 100% methanol with 5 mM ammonium acetate. Data were acquired in broadband collision-induced dissociation (bbCID). QA/QC included procedural blanks, surrogate recoveries (70–120%), and calibration standards to ensure method performance as described elsewhere.^20,21^

A target screening using the TASQ software 2023b (Bruker) was performed for 25 compunds, 1 antifungal (miconazole) and 24 antibiotics, which were representatives of various therapeutic classes, including beta-lactams, diaminopyrimidines, fluoroquinolones, lincosamides, macrolides, phenicols, rifamycins, sulfonamides, and tetracyclines (Supporting Information Table S2). After, data were exported and corrected with blank subtraction and surrogate recovery. Surrogates were assigned to detected compounds based on antibiotic families and retention times. See Supporting Information Tables S1 and S2 for information on antibiotic surrogate mix and targeted compounds, respectively.

To calculate the removal efficiencies for each antibiotic we used eq 1.

1 removalefficiency(%)=(1−effluentconc./influentconc.)×100

Equation used for quantifying the removal efficiency for antibiotics.

### 2.3 Metagenomic Sequencing and Processing

#### 2.3.1 DNA Extraction, Library Preparation, and Sequencing

Water samples were collected in sterile 2 L glass containers, maintained at 4 °C, and filtered within 24 h of arrival at the laboratory. For each sampling point, three biological replicates were collected for influent, effluent, and sludge samples over three consecutive weeks. Filtration was performed through 0.22 μm nylon membrane filters (Whatman, Maidstone, UK) using a filtration device and a vacuum pump until membrane saturation. Membrane saturation was operationally defined as the point at which further filtration was no longer feasible due to clogging and a marked decrease in flow rate. Under these conditions, filtration volumes corresponded to approximately 35 mL for influent samples and 200 mL for effluent samples. Filters were then stored at −20 °C until DNA extraction. 250 mg of sludge was weighed using a precision scale (Mettler Toledo, MS-S/MS-L model), transferred into sterile Eppendorf tubes, and stored at −20 °C until DNA extraction. Extraction of DNA from filters and sludge samples was performed using the DNeasy PowerSoil kit (Qiagen Laboratories, Inc.) according to the manufacturer’s instructions. The concentration and the quality of the DNA was tested using a NanoDrop Spectrophotometer 8000 (Thermofisher Scientific, Inc.), and all of the DNA extracts were stored at −20 °C until DNA library preparation. All 27 DNA samples were sent to Novogene UK for DNA library preparation with the NEBNext Ultra IIDNA Library Prep Kit (Cat No. E7645; New England Biolabs), and for paired-end 150 bp sequencing on a single lane on a NovaSeq X Plus Series platform (Illumina) using the 25B flow cell. No host DNA depletion was performed before DNA library preparation.

#### 2.3.2 Bioinformatics and Statistical Analysis

Raw sequence data were uploaded to the sequence read archive (SRA) within the National Center for Biotechnology Information (NCBI) database and made available under the BioProject accession number PRJNA1329243. To quality-control and preprocess the raw sequence reads, and to align the resulting preprocessed reads to reference databases, the BaitCapture Nextflow workflow was used.^22^ For versions of software packages, databases, and metadata files used, and for bioinformatics parameters, see Supporting Information Table S3. All metagenomic analyses were performed using unassembled reads, and no de novo assembly was conducted. First, the overall quality of the raw paired-end sequence reads was assessed with FastQC and MultiQC.^23,24^ Next, the paired-end sequence reads were preprocessed using fastp with default parameters to filter low-quality reads and reads shorter than 15 bp, and with a provided FASTA file of Illumina TruSeq adapter sequences to remove adapter contamination.^25^ The preprocessed reads were then aligned against the nucleotide homologue Comprehensive Antibiotic Resistance Database (CARD) and MobileGeneticElementDatabase (from https://github.com/KatariinaParnanen/MobileGeneticElementDatabase) using KMA with nondefault options;^26–28^ briefly, each query sequence was forced to match to only one target sequence, the accumulated alignment algorithm used mapping scores instead of alignment scores to optimize memory usage, and all *k*-mers were searched exhaustively during mapping. Finally, the sequence coverage and depth statistics from the alignments were obtained using custom R scripts, Mosdepth, and SAMtools.^29–31^

The clinical significance of identified ARGs was assessed using the risk ranking framework developed by Zhang et al.,^32^ as implemented in the Structured ARG Database (SARG). Briefly, this framework classifies ARGs into four risk categories (Ranks I–IV) based on three criteria, namely: (i) enrichment in human-associated environments, (ii) association with mobile genetic elements, and (iii) presence in clinically relevant pathogens. ARGs meeting all three criteria are classified as Rank I (highest risk), whereas those failing one or more criteria are assigned to lower risk categories (Ranks II–IV).

To apply the AMR risk classification framework developed by Zhang et al.,^32^ to our metagenomic data, reference protein sequences corresponding to CARD nucleotide targets were matched to reference proteins within the SARG. Specifically, reference protein sequences from the CARD protein homologue database were aligned to the SARG protein database using BLASTp, and only perfect alignments were retained for matching CARD targets to SARG targets. Risk ranking metadata associated with matched SARG entries were retrieved from the SARG risk ranking resource (https://smile.hku.hk/ARGs/Indexing/riskranking) and subsequently assigned to the corresponding CARD ARGs. CARD reference ARGs without an assigned risk level (32.4% of targets) either (i) did not perfectly match any ARG within SARG or (ii) perfectly matched an ARG within SARG for which no risk level had been assigned. These ARGs were retained in the data set but were not classified as high-risk. The list of CARD ARGs with identical SARG matches and associated risk rankings is provided in Supporting Information Table S4.

The number of ARGs and MGEs per prokaryotic cell were estimated by dividing the total fold-coverages of all gene targets by the mean fold-coverages of ribosomal essential single-copy marker genes (ESCMGs) for prokaryotes, similar to the approach used previously.^33^ In summary, a list of prokaryotic ESCMGs^34^ was filtered for only ribosomal gene targets (*n* = 27; Supporting Information S1), and alleles corresponding to these ribosomal ESCMGs were extracted from the PubMLST ribosomal multilocus sequence typing (MLST).^35^ The resulting alleles (*n* = 3,363,580) were reduced to a nonredundant set of ribosomal ESCMG alleles with CD-HIT at a 95% sequence identity threshold (options: -c 0.95 -aS 0.95), and the preprocessed wastewater sequence reads were aligned against the remaining nonredundant alleles (*n* = 1,459,782) with KMA, using the same options as above, to obtain mean fold-coverages of ribosomal ESCMGs for each sample. The presence of a single prokaryotic cell should theoretically correspond to a mean ribosomal ESCMG fold-coverage of 1, so normalizing the fold-coverages of ARGs and MGEs by this value provides an estimate of the number of gene targets per prokaryotic cell.

The bacterial community composition of the wastewater samples was determined with the nf-core/taxprofiler Nextflow workflow.^36^ Raw paired-end sequence reads were preprocessed using the same parameters and version of fastp as described above for the BaitCapture workflow. The latest version of the ChocoPhlAnSGB database was downloaded using the metaphlan --install command within MetaPhlAn4, and this database, along with the preprocessed sequence reads, were used as input to nf-core/taxprofiler for taxonomic analysis with MetaPhlAn4.^36^ Afterward, the resulting bacterial taxa relative abundances were converted to estimated taxa counts using the *.metaphlan.bowtie2out.txt files generated by MetaPhlAn, and the ChocoPhlAnSGB-based taxonomies were converted to Genome Taxonomy Database (GTDB)-based taxonomies using the MetaPhlAn-provided mpa_vJun23_CHOCOPhlAnSGB_202307_SGB2GTDB.tsv taxonomy mapping file and sgb_to_gtdb_profile.py script. Phyla named “Bacillota”, “Bacillota_A”, “Bacillota_B”, and “Bacillota_C” were merged into a single Bacillota phylum for downstream analysis, and *Proteobacteria*, *Cyanobacteria*, and *Patescibacteria* were renamed to *Pseudomonadota*, *Cyanobacteria*, and *Patescibacteriota* to reflect recent updates to the International Code of Nomenclature of Prokaryotes.

Differentially abundant ARGs, MGEs, and bacterial taxa were identified using the Analysis of Composition of Microbiomes with Bias Correction (ANCOM-BC) software implemented in R with nondefault options to use Bonferroni correction for multiple comparisons, keeping all taxa, performing a global test, and using a conservative variance estimate of the test statistic. For each test, the influent samples were used as the reference group. ANCOM-BC reports log fold-change (LogFC), standardized log fold-change (LogFC_std_) by mean difference, and adjusted *P* values: targets or taxa were classified as differentially abundant if |LogFC_std_| ≥ 10 and adjusted *P* ≤ 0.05, while LogFC values are reported in text and figures as log_2_ fold-changes (Log2FC) for easier interpretability. For reference, a Log2FC of 1 corresponds to a 2-fold increase (doubling), Log2FC of 2 corresponds to a 4-fold increase (quadrupling), and Log2FC of −1 corresponds to a 2-fold decrease (halving).

The alpha diversity of ARGs, MGEs, and bacterial taxa was assessed using observed richness computed manually, and Simpson’s index and Chao1 richness estimator diversity index computed by the microbiome package implemented in R.^37^ For more conservative AMR reporting, ARGs and MGEs (but not bacterial taxa) were considered to be present in a sample and contributing to observed richness only if the proportion of the reference target sequence length covered by aligned reads was ≥0.7 and the fold-coverage of aligned reads against the reference target sequence was ≥0.7; otherwise, they were considered to be absent. All aligned reads were used for computing beta diversity. Beta diversity was assessed by performing a center log ratio (CLR) transformation of target fold-coverages and estimated taxa counts to obtain Aitchison distances (CLR-transformed relative abundances), and a log2-based transformation was used so that differences in Aitchison distances would represent fold-changes. Differences in the composition of ARGs, MGEs, and taxa by sample type and WWTP were evaluated by performing a principal component analysis (PCA) of Aitchison distances, and differences in composition due to antibiotic residue concentrations were evaluated by performing a redundancy analysis (RDA) using the microViz R package.^38^ Only antibiotic residues that were detected in at least one sample were included in the RDA. Collinear concentrations of antibiotic residues were excluded from the RDA based upon a 0.9 pairwise absolute correlation cutoff using the find correlation function within the caret package implemented in R.^39^

All statistical tests were performed in R and all data visualizations were created using the ggplot2 package unless otherwise specified.^40^ Averages are reported as mean ± standard deviation (S.D.). Global differences between group medians were evaluated using a Kruskal–Wallis test from the base R stats package, followed by a post hoc Dunn’s test for pairwise differences from the rstatix package as a wrapper within the ggpubr package. The false-discovery rate (FDR) was controlled by Bonferroni correction during multiple hypothesis testing.^41,42^ All *p* values are FDR-adjusted and reported with a significance threshold of *p* < 0.05. Differences in the dispersion of gene and taxa abundances in the PCAs were evaluated using a permutational multivariate ANOVA (PERMANOVA) with the *adonis2* function from the vegan package, implemented as a wrapper within the microViz package, and using 9999 permutations, a seed of 123, and WWTP and sample type as predictor variables.^43^ Cross-associations between ARG and bacterial taxa abundances were assessed by calculating a Spearman’s rank correlation matrix from the Aitchison distances, and network plots of positive correlations were visualized using the ggraph and igraph packages.^44,45^

The significance of antibiotic residue concentrations for explaining variation in gene and taxa abundances within the RDAs was assessed using the *anova.cca* function within vegan with 999 permutations, after controlling for site-specific differences by using WWTP as a conditioning variable. For the purpose of comparing antibiotic residue concentrations from influent and effluent samples (measured in ng/L) with sludge samples (measured in ng/g) in the RDAs, we assumed a bulk sludge density of 1.01 kg/m^3^. Heatmaps were created using the ggalign and ComplexHeatmap packages, and taxonomy barplots were created using the microViz and microshades packages.^46^

## 3 Results

### 3.1 Antibiotic Detection, Concentration, and Removal in Wastewater and Sludge

In water sample, of the 25 compounds analyzed (Supporting Information Table S2), 10–11 compounds were consistently detected in influents and 8–11 in effluents. Detection frequencies were highest in P.LL, followed by F.LL and G-V (Supporting Information Figure S1). The detected antibiotics were fluoroquinolones (ciprofloxacin, ofloxacin, levofloxacin and enrofloxacin,), macrolides (azithromycin and clarithromycin), sulfonamides (sulfamethoxazole and acetyl-sulfamethoxazole), tetracyclines (doxycycline), and others such as trimethoprim and rifaximin (Supporting Information Table S5). Influent concentrations ranged from ∼70 to >4000 ng/L, with doxycycline and rifaximin among the most abundant (>3000 ng/L in P.LL and F.LL). Effluent levels remained substantial for several compounds, including ciprofloxacin, ofloxacin, levofloxacin, clarithromycin, sulfamethoxazole, and azithromycin, often in the hundreds of ng/L (Supporting Information Table S5). In some cases (e.g., trimethoprim in F.LL), effluent concentrations exceeded influent levels, suggesting deconjugation of conjugated metabolites.

In sludge, of the 25 compounds analyzed (Supporting Information Table S2), 8–9 compounds were detected (Figure S1). The main families of compounds detected were fluoroquinolones (ciprofloxacin, ofloxacin, levofloxacin, enrofloxacin and norfloxacin), macrolides (azithromycin), tetracyclines (doxycycline), rifamycins (rifaximin) and other such as miconazole (Supporting Information Table S6). Concentrations of antibiotic residues in sludge ranged from <10 ng/g to nearly 1 μg/g. Fluoroquinolones clearly dominated, with ciprofloxacin peaking at ∼965 ng/g in G-V sludge, followed by ofloxacin and levofloxacin. In contrast, tetracyclines such as doxycycline were present only at low levels (<30 ng/g), consistent with their degradation under anaerobic digestion. Miconazole was detected sporadically and generally near the limit of quantification (LOQ) (Supporting Information Table S6). Overall, fluoroquinolones and macrolides persisted across matrices, and several antibiotics exceeded their Predicted no-effect concentrations (PNECs) in effluents, indicating sustained selective pressure for resistance. PNEC values were obtained from Bengtsson-Palme and Larsson.^47^

Removal efficiencies in effluents varied substantially among antibiotics and across WWTPs, ranging from <30% to >95% (Table 1). The highest removal rates across the three WWTPs were observed for acetyl-sulfamethoxazole (97–100%), rifaximin (90–99%), and enrofloxacin (86%, only quantified in P.LL). Acetyl-sulfamethoxazole was completely removed only in F.LL (100 ± 0%). In contrast, a removal efficiency of 40% or less was observed in P.LL for sulfamethoxazole (34%) and trimethoprim (11%), in F.LL for clarithromycin (27%) and levofloxacin (22%), and in G-V for doxycycline (22%) and sulfamethoxazole (40%) (Table 1).

**Table 1:** Percentage of Removal Efficiency Expressed as Fraction of Influent Concentration Removed (Mean ± Standard Deviation) of Antibiotics Detected in Effluents of the Three WWTP: Gavà- Viladecans (G-V), El Prat de Llobregat (P.LL), and Sant Feliu de Llobregat (F.LL), *N* = 3. ND; Not Detected. Negative Removal Values Indicate a Net Increase in Concentration between Influent and Effluent

antibiotics	P.LL	F.LL	G-V
acetyl-sulfamethoxazole	97 ± 1	100 ± 1	97 ± 1
azithromycin	64 ± 17	71 ± 16	66 ± 6
ciprofloxacin	79 ± 4	45 ± 1	61 ± 10
clarithromycin	68 ± 17	27 ± 22	0.47 ± 15
doxycycline	60 ± 8	49 ± 3	22 ± 7
enrofloxacin	86 ± 5	ND	ND
levofloxacin	61 ± 7	22 ± 7	47 ± 5
ofloxacin	71 ± 10	63 ± 32	81 ± 4
rifaximin	95 ± 5	90 ± 17	99 ± 1
sulfamethoxazole	34 ± 12	51 ± 6	41 ± 18
trimethoprim	11 ± 3	(−)15 ± 3	54 ± 3

### 3.2 Impact of the Treatment on the Composition of Bacterial Communities

In total, 27 metagenomes were analyzed. Metagenomic sequencing yielded 1.8 B paired-end reads and 268.1 Gbp across all samples, with a mean of 66.2 M reads per sample (range: 52.1–79.7 M).

Across the three WWTPs, the influent samples were dominated by *Pseudomonadota*, followed by *Bacillota*, *Actinobacteriota*, *Bacteroidota* and *Campylobacterota*, with *Verrucomicrobiota*, *Desulfobacterota*, and *Fusobacteriota* also present at lower relative abundances (Figure 1). Several of these phyla, including *Fusobacteriota*, *Verrucomicrobiota*, *Bacillota*, *Bacteroidota*, and *Pseudomonadota*, showed some of the largest decreases in abundance in the effluent samples (Figure 1, Supporting Information Figure S2). *Pseudomonadota*, *Bacillota*, *Actinobacteriota*, *Bacteroidota* and *Campylobacterota* remained the dominant phyla in the effluents; however, differences in the relative abundance were observed between WWTPs, particularly between P.LL and F.LL compared to G-V (Figure 1). G-V exhibited higher relative abundances of *Pseudomonadota* and *Actinobacteriota* but lower abundances of *Bacteroidota*, *Bacillota* and *Campylobacterota* relative to P.LL and F.LL. Additionally, G-V showed increased abundances of *Nitrospirota* and *Deinococcota*, whereas P.LL and F.LL were enriched in *Spirochaetota*. In sludge samples, clear differences were observed among the three WWTPs. The P.LL sludge was dominated primarily by *Actinobacteriota*, followed by *Pseudomonadota* and *Choroflexota*. In contrast, F.LL exhibited a more diverse composition, with *Choroflexota*, *Actinobacteriota*, *Pseudomonadota*, and *Bacteroidota* as the most abundant phyla. G-V sludge was dominated by *Bacteroidota*, *Actinobacteriota*, and *Pseudomonadota*, along with a notable presence of *Cloacimonadota* (Figure 1). These patterns were reflected in the PCA (Supporting Information Figure S3C), which revealed three distinct compositions according to sample type.

**Figure 1:**
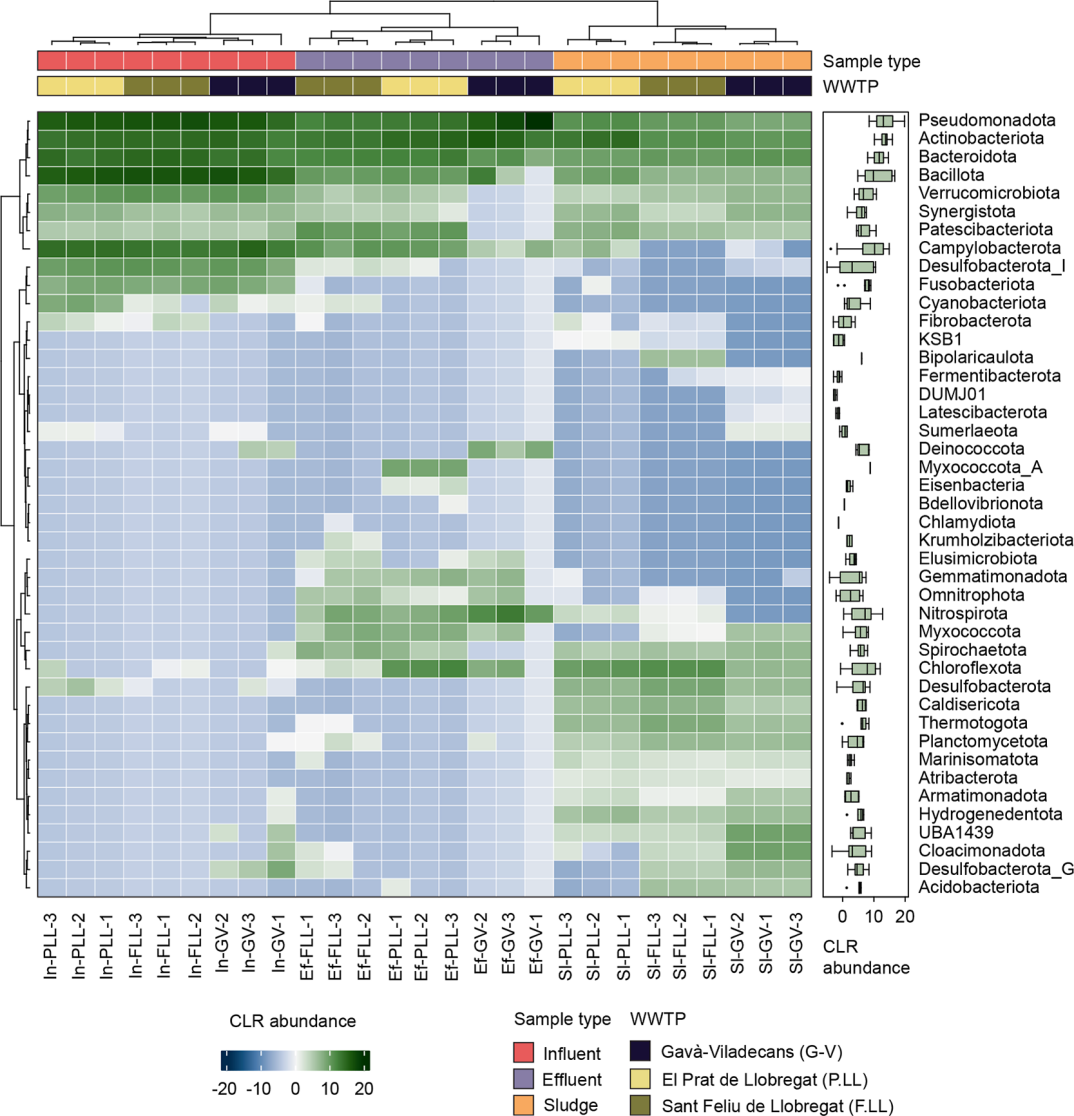
Heatmap of center log ratio (CLR)-transformed bacterial phylum relative abundances. Hierarchical clustering of samples and phyla was performed using a Euclidean distance matrix with ward.D agglomeration. Distributions of CLR-transformed abundances of phyla across all samples are shown by boxplots on the right. Samples are from the Prat de Llobregat (P.LL), Sant Feliu de Llobregat (F.LL), and Gavà-Viladecans (G-V) WWTPs.

At the family level, influent profiles across the three WWTPs were similar and dominated by *Burkholderiaceae* and *Moraxellaceae* (*Pseudomonadota*), *Lachnospiraceae*, *Ruminococcaceae*, and *Streptococcaceae* (*Bacillota*), and *Bacteroidaceae* (*Bacteroidota*) (Supporting Information Figure S4). Effluents differed by plant. P.LL and F.LL showed higher proportions of *Actinobacteriota*, especially *Microtrichaceae* and an unclassified *Actinobacteriota* lineage (UBA10799), with *Mycobacteriaceae* also present. These two plants reduced *Bacillotafamilies* but retained proportionally more *Bacillota* and *Bacteroidota* than G-V, including *Tannerellaceae* and some *Bacteroidaceae*. In contrast, G-V showed very low *Bacillota* and *Bacteroidota* and a marked increase in *Rhodobacteraceae* (*Pseudomonadota*) (Supporting Information Figure S4). Sludge bacterial communities were slightly different among WWTPs. P.LL sludge was largely dominated by *Microtrichaceae* (*Actinobacteriota*), similar to its effluent. In contrast, F.LL, and especially G-V, had higher proportions of *Bacteroidaceae* and *Tannerellaceae* than P.LL. Across plants, *Bacillota* and *Pseudomonadota* were present at broadly comparable levels (Supporting Information Figure S4).

### 3.3 Richness and Abundance of ARGs and MGEs

In total, 561 unique ARGs and 1293 unique MGEs were detected across all samples. On average, 317 ± 17 ARGs and 860 ± 27 MGEs (at the reference sequence level) were detected in influent samples. In the effluent, levels decreased to 95 ± 23 ARGs and 382 ± 94 MGEs, while sludge contained 80 ± 15 ARGs and 309 ± 45 MGEs. The Chao1 diversity index followed a similar trend across sample types (Supporting Information Figure S5, left panels), with the lowest values observed in G-V effluents (Supporting Information Figure S5, right panels). In contrast, Simpson’s evenness index increased significantly in effluents relative to influent samples, indicating a less diverse but more homogeneous ARG and MGE distribution (Supporting Information Figure S6A,B). The total relative abundance, expressed as the number of ARGs and MGEs per cell, was significantly higher in the influent, with means of 1.00 ± 0.03 and 7.0 ± 1.12, respectively, compared to 0.30 ± 0.03 and 1.10 ± 0.60 in the effluent (Dunn’s test, *p* < 0.05) and 0.20 ± 0.01 and 0.7 ± 0.10 in the sludge (*p* < 0.0001) (Figure 2A,B). The abundance of ARGs per cell was comparable among the three WWTPs, except in sludge samples, where P.LL exhibited significantly higher values than G-V (Dunńs test, *p* < 0.05) (Supporting Information Figure S7). A similar trend was observed for MGEs in sludge samples. In the effluent, G-V displayed a lower abundance of MGEs compared with P.LL (*p* < 0.05) (Supporting Information Figure S7).

**Figure 2:**
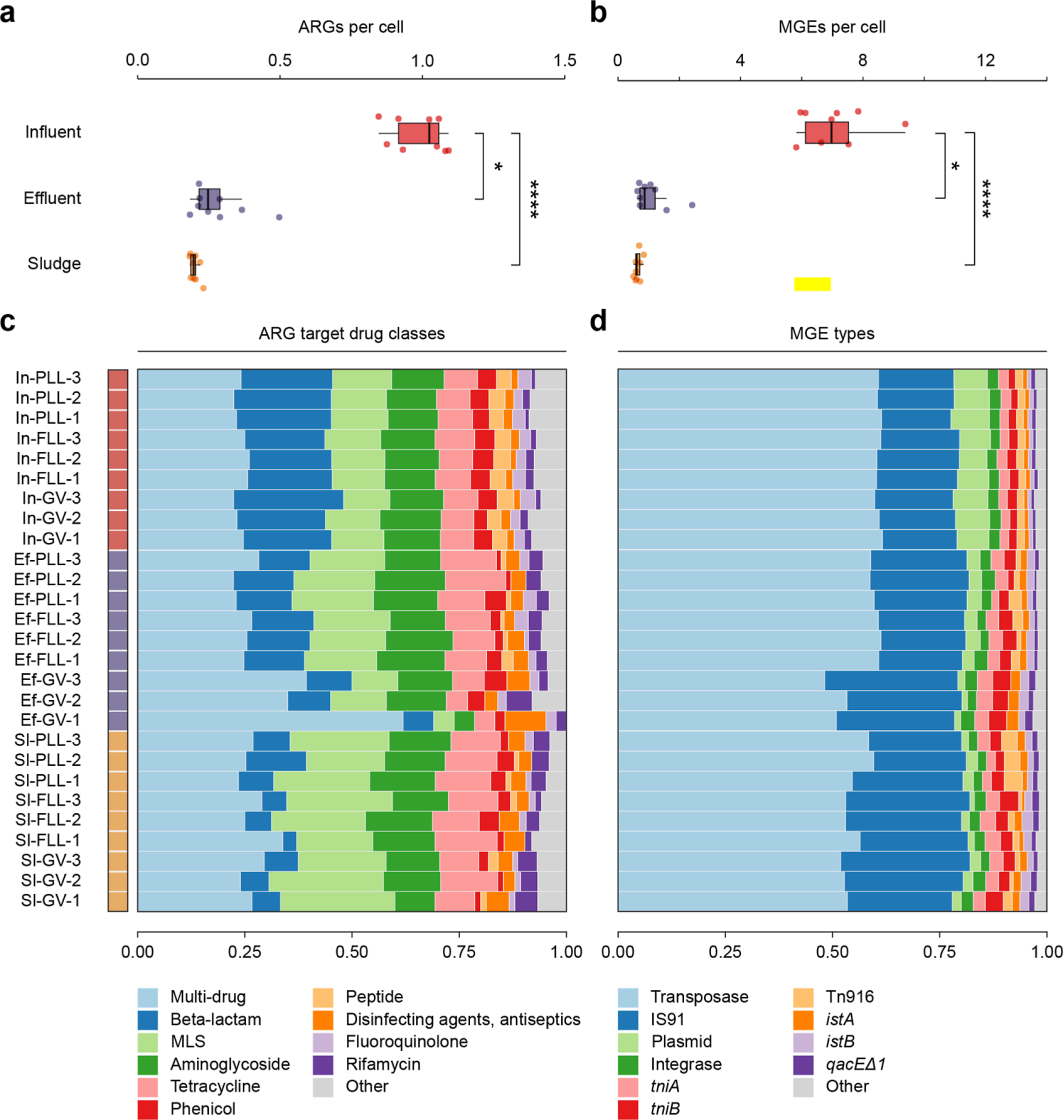
Resistome and mobilome profiles of influent, effluent, and sludge samples from the Prat de Llobregat (P.LL), Sant Feliu de Llobregat (F.LL), and Gavà-Viladecans (G-V) WWTPs. Total abundance is shown in boxplots as (A) ARG and (B) MGE copies per prokaryotic cell, with data pooled from all three WWTPs (*n* = 9 per sample type), while composition is shown in stacked barplots of the top 10 most prevalent ARG target drug classes (C) and MGE types (D). Target detection thresholds (outlined in Methods), were used to determine if an ARG or MGE was present or absent. Dunn’s test with Bonferroni correction was used for posthoc pairwise comparisons. *, *p* < 0.05; **, *p* < 0.01; ***, *p* < 0.001; ****, *p* < 0.0001.

### 3.4 Impact of the Treatment on the Resistome and Mobilome Composition

The most prevalent ARG target drug classes in the influent, on average, were multidrug (24.1 ± 1.4) and beta-lactams (21.2 ± 2.1%), followed by macrolide-lincosamide-streptogramin (MLS) (12.6 ± 0.9%), aminoglycosides (12.5 ± 0.8%), and tetracyclines (8.1 ± 0.5%) (Figure 2C). The composition was relatively consistent across WWTPs, showing minimal variation. In the effluent, multidrug (32% ± 12.6), MLS (15.1 ± 4.8%), aminoglycosides (13.4 ± 3.5%), beta-lactams (12.2 ± 2.6%) and tetracyclines (9.5 ± 3.2%) remained dominant, but clear WWTP-level differences emerged: G-V had notably lower beta-lactams (9.3 ± 1.9%), MLS (9.5 ± 4.2%) and tetracyclines (5.7 ± 1.5%) compared to F.LL and P.LL, while multidrug resistance genes were proportionally higher in G-V (45.4 ± 14.4%) than in the other plants. In sludge samples, multidrug resistance genes were the most abundant class (27.1 ± 3.3%), followed by MLS (22.4 ± 3.3%), aminoglycosides (13.6 ± 1.9%), and tetracyclines (11.7 ± 1.8%), with only minor differences observed among WWTPs (Figure 2C). These differences between WWTPs were clearly reflected in the PCA, where influent samples clustered together without distinction among WWTPs and were significantly separated from effluent and sludge samples (PERMANOVA, *p* < 0.05) (Supporting Information Figure S3A). Effluent samples from P.LL and F.LL clustered together and were segregated from the G-V effluent samples.

The most prevalent MGEs in the influent mobilome (total MGEs), on average, were transposases (60.8 ± 0.6%) and insertion element IS91 (18.0 ± 0.9%), followed by plasmids (7.7 ± 0.7%), and integron integrases (2.5 ± 0.1%) (Figure 2D).

#### 3.4.1 ARGs with High Risk in Reclaimed Effluents

Hundreds of ARGs have been identified in environmental waters, posing varying levels of health risk.^48,49^ Accordingly, we opted to classify the ARGs detected in influent, effluent, and sludge samples into four risk categories (Rank I–IV) based on the framework by Zhang et al.,^48^ which integrates anthropogenic prevalence, gene mobility, and the pathogenic potential of its bacterial hosts. In total, 4, 19, and 20 out of 43 unique Rank I ARGs were detected in reclaimed effluents from Barcelona, representing 3%, 14%, and 11% of the total ARGs richness in G-V (*n* = 126), P.LL (*n* = 133) and F.LL (*n* = 177) effluents, respectively. As shown in Table 2, risk I ARGs were efficiently removed, especially in G-V, where effluent detection declined by >90%, followed by P.LL (59%) and F.LL (48%). In contrast, effluent detection of Risk IV ARGs was reduced by 64% in G-V, 75% in P.LL, and 69% in F.LL. Overall, G-V showed the highest removal for high-risk ARGs, while P.LL and F.LL performed slightly better in reducing low-risk ARGs (Table 2). Among the Rank I ARGs, the most abundant genes in influent samples across the three WWTPs were those predicted to confer resistance to aminoglycosides (e.g., *aadA6, aadA5*, *aadA8*, *aph* variants), macrolides (*ermB*, *mphA*), lincosamides (*lnuB*), phenicols (*catB3*), and macrolide esterases (*ereA2*) (Figure 3). Despite treatment, several of these highly abundant genes remained detectable in effluent samples from all three plants, indicating limited removal efficiency. Notably, genes *aadA5, aadA6, aadA8*, *aph*(3′’)*-Ib*, *ermB*, and *mphA* were consistently detected at elevated levels in final effluents and sludge samples (Figure 3). In addition, *bla*_OXA‑129_, a class D beta-lactamase gene associated with resistance to penicillins and cephalosporins, was detected at higher abundance in the influent of F.LL compared to G-V and P.LL. In the effluent, this gene persisted in F.LL and P.LL, and at a much lower abundance in G-V; however, *bla*_OXA‑129_ was more abundant in the sludge of G-V compared to the other two WWTPs (Figure 3). The gene *sul3* was detected in all influent samples, partially removed in effluents, but enriched in sludge, suggesting it could serve as a candidate marker for monitoring in sludge applied to agriculture.

**Table 2:** Mean Number of ARGs Detected (±SD) Across Four Risk Categories (Rank I–IV) in Influent, Effluent, and Sludge Samples from Three WWTPs in Barcelona: Gavà-Viladecans (G-V), El Prat de Llobregat (P.LL), and Sant Feliu de Llobregat (F.LL). ARG risk categories were assigned following the framework by Zhang et Al. (2021). Only ARGs that met target detection thresholds (outlined in methods) were counted.

risk_Level	G-V	P.LL	F.LL	G-V	P.LL	F.LL	G-V	P.LL	F.LL
	influent			effluent			sludge		
I	29 ± 1.0	34 ± 1.2	27 ± 0.6	2 ± 2.0	14 ± 2.4	14 ± 2.7	11 ± 0.6	13 ± 1.6	8 ± 2.0
II	16 ± 1.2	15 ± 1.0	15 ± 1.0	3 ± 2.6	8 ± 1.0	10 ± 0.6	4 ± 0.6	4 ± 1.0	3 ± 0.6
III	59 ± 4.1	60 ± 1.0	49 ± 3.0	2 ± 1.0	10 ± 1.2	15 ± 4.1	7 ± 1.0	12 ± 1.6	7 ± 0.6
IV	145 ± 14.2	154 ± 5.0	145 ± 4.7	52 ± 22.6	39 ± 2.6	45 ± 4.6	39 ± 5.6	45 ± 6.1	34 ± 1.6

**Figure 3:**
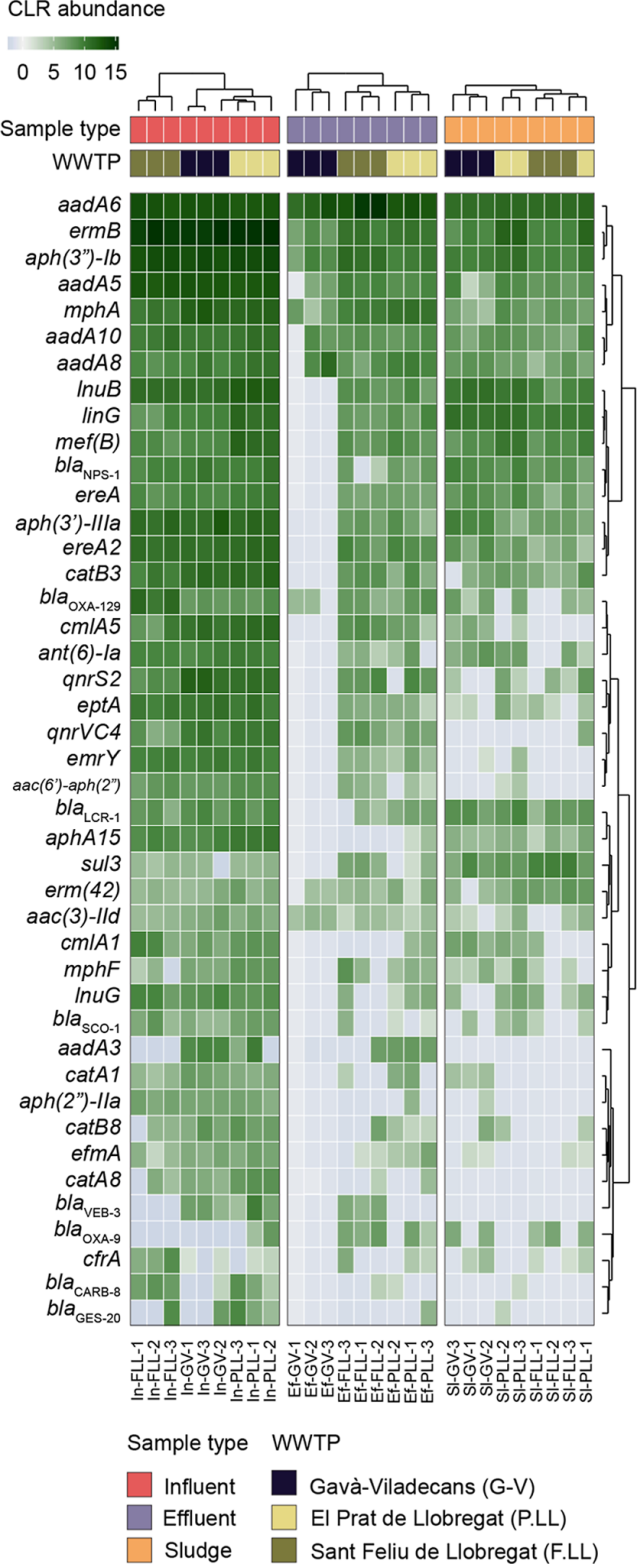
Center log ratio-transformed relative abundances of Risk Level 1 ARGs in the three WWTPs: Gavà- Viladecans (G-V), El Prat de Llobregat (P.LL), and Sant Feliu de Llobregat (F.LL). Hierarchical clustering of samples and targets was performed using a Euclidean distance matrix with ward.D agglomeration. Only ARGs that met the positive target detection thresholds for at least one sample are displayed (see Methods for detection criteria). ARG risk categories were assigned following the framework by Zhang et al.^48^

### 3.5 Removal Efficiency of ARGs and MGEs in Reclaimed Effluents and Sludge Samples

#### 3.5.1 ARG Removal is Largely Drug-Class Dependent

When comparing effluent to influent, certain ARG drug classes displayed consistent patterns across all three WWTPs despite their different treatment configurations, suggesting that their fate is largely independent of plant-specific processes (Figure 4A). Genes predicted to confer resistance to diaminopyrimidines were consistently removed, with the strongest reduction in G-V (3.7-fold decrease). Similarly, genes predicted to confer resistance to phosphonic acid, nucleosides, nitroimidazoles, and MLS were also consistently removed, with the highest reductions observed in G-V and F.LL. In turn, genes predicted to confer resistance to aminoglycosides, fluoroquinolones, glycopeptides, and beta-lactams showed no substantial change in any WWTP (Figure 4A). Consistent enrichment across plants was observed for elfamycin and sulfonamide resistance genes, both with the highest enrichment in P.LL (4.2-fold increase and 7.0-fold increase, respectively). Rifamycin and bicyclomycin ARGs showed similar enrichment across WWTPs (3.8-fold increase and 4.7-fold increase on average, respectively), while fusidane was enriched in all plants but to the greatest extent in F.LL (10.8-fold increase) (Figure 4A).

**Figure 4:**
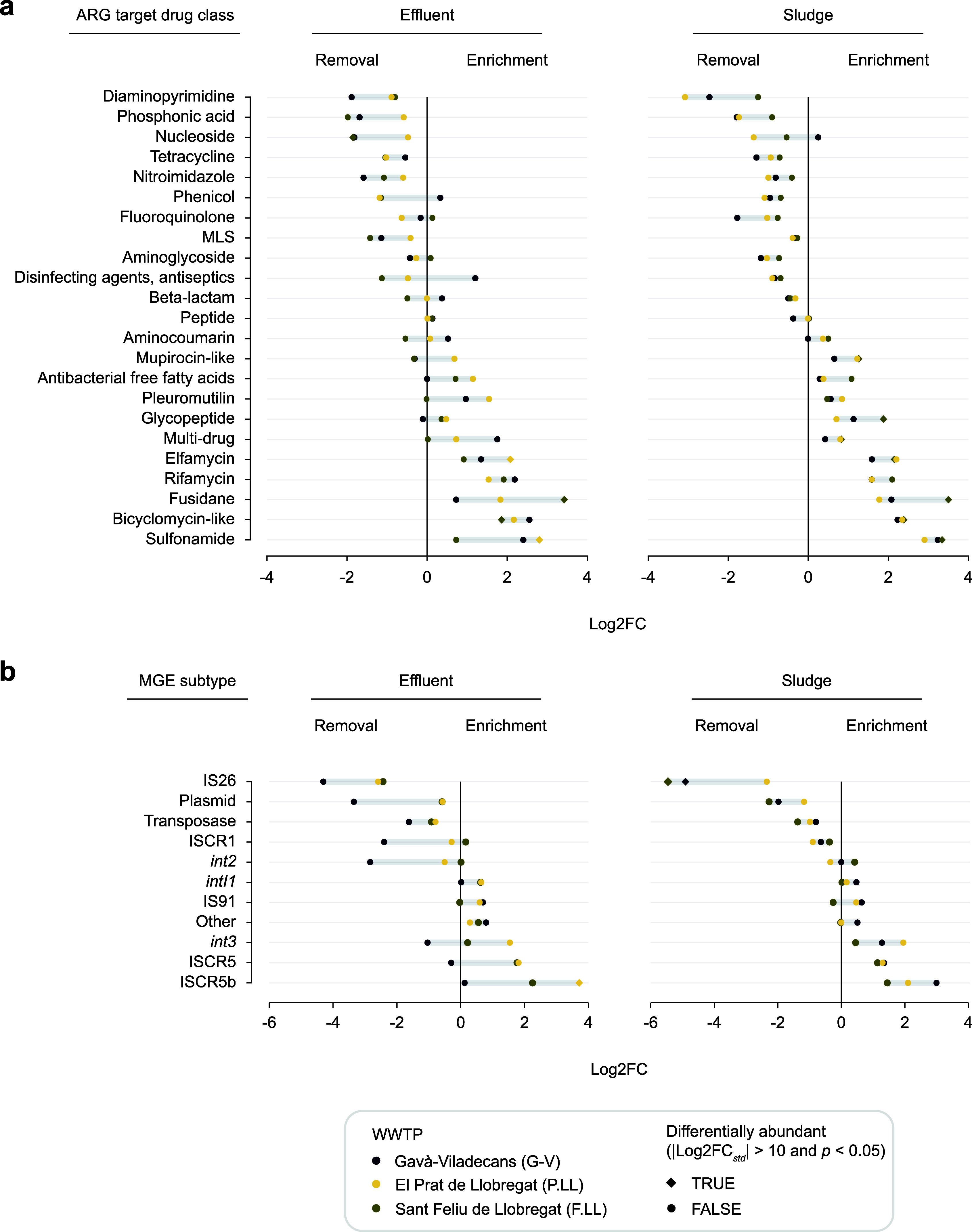
Removal efficiency of ARGs by target drug class (A) and selected MGE subtypes (B) expressed as fold-changes, shown on the *x*-axis in Log2 scale (Log2FC). Positive Log2FC values indicate enrichment while negative values indicate removal. For reference, a Log2FC of 1 corresponds to a 2-fold increase (doubling), a Log2FC of 2 to a 4-fold increase (quadrupling), and a Log2FC of −1 to a 2-fold decrease (halving). Significantly differentially abundant classes or elements are indicated by diamond symbols. ARG drug classes and MGE subtypes are considered differentially abundant if | Log2FC_std_| > 10 and *p* < 0.05.

In contrast, other ARG classes exhibited clear plant-specific patterns, indicating that removal or enrichment may be influenced by the type of treatment applied. Phenicol and disinfectant ARG classes were reduced in P.LL and F.LL but enriched in G-V, reaching 1.3- and 2.3-fold increases, respectively. Pleuromutilins and multidrug ARGs were enriched in both P.LL (- and 1.7-fold increases, respectively) and G-V (2.0- and 3.4-fold increases), but not in F.LL. These differences highlight that, while some ARG classes respond similarly to biological wastewater treatment regardless of configuration, others appear more sensitive to plant-specific operational conditions. A similar pattern was observed in sludge samples, where the drug classes reduced in the effluent also tended to be reduced in the sludge, and those enriched in the effluent were likewise enriched in the sludge (Figure 4B).

#### 3.5.2 Removal of MGEs in Effluents Depends on WWTP Treatment

Ten MGEs or mobile genetic element–associated markers commonly detected in pathogenic bacteria and central to the dissemination of AMR were selected to explore the removal efficiency of the three WWTP studied (Figure 4B). These include plasmids (identified by replication initiation genes and colicins), transposable elements such as insertion sequences (IS26 and IS91), insertion sequence common region elements (ISCR1, ISCR5, and ISCR5b), transposases, and integron integrases (*intI*1, *intI*2, and *intI*3).^14,15,17,50–54^

In the effluent, IS26 and plasmids were consistently removed across all WWTPs, with G-V showing the greatest reductions (19.8-fold decrease for IS26 and 10.2-fold decrease for plasmids) compared to P.LL and F.LL, which showed similar removal levels (IS26:5.71 ± 0.43-fold decrease; plasmids: 1.49 ± 0.01-fold decrease) (Figure 4B). In contrast, *intI1* and IS91 showed no substantial removal in any WWTP, with fold-changes close to or above 1, indicating persistence. Some MGEs exhibited WWTP-specific removal: ISCR1 and *intI2* were strongly reduced in G-V (5.3 and 7.1-fold decrease, respectively) but not in P.LL or F.LL, where changes were negligible or even positive. Conversely, *intI3*, ISCR5, and ISCR5b were enriched in P.LL and F.LL (up to 3.5-fold for ISCR5) but remained stable or slightly reduced in G-V (Figure 4B). These patterns indicate that, among the three WWTPs, G-V consistently achieved the highest removal efficiency for these pathogen-associated MGEs in the effluent stage.

In sludge samples (Figure 4B), removal patterns were generally consistent across WWTPs, with fewer interplant differences compared to the effluent. The main exception was IS26, which showed greater removal in G-V and F.LL than in P.LL. Overall, the same broad trends observed in the effluent were maintained: certain MGEs, such as IS26 and plasmids, were consistently reduced; others, including *intI1* and IS91, persisted with little or no change; while a subset, such as i*ntI3*, ISCR5, and ISCR5b, exhibited enrichment (Figure 4B). This suggests that the fate of many MGEs during treatment is largely independent of WWTP-specific processes in the sludge stage.

### 3.6 Associations between Selected Risk Level I ARGs and Bacterial Taxa

To identify potential carriers of Risk Level I ARGs that withstand wastewater treatment, a cross-association analysis between selected ARGs (*ermB*, *aadA5*, *aadA6*, *aadA8*, *aph­(3*”*)*-*Ib*, *bla*_OXA‑129_) and bacterial taxa abundances was performed at different taxonomic levels.

At the family level, *ermB*, *aph*(3”)-*Ib*, and *aadA5* showed strong correlations with taxa associated with human pathogens, including *Enterobacteriaceae*, *Streptococcaceae*, *Enterococcaceae*, *Neisseriaceae*, *Clostridiaceae*, and *Peptostreptococcaceae* (Spearman’s *p* > 0.7, *p* < 0.05). These ARGs were also strongly correlated with families comprising opportunistic pathogens or human commensals known to act as reservoirs of antimicrobial resistance, such as *Aerococcaceae*, *Arcobacteraceae*, *Bacteroidaceae*, *Bifidobacteriaceae*, *Filifactoraceae*, *Lachnospiraceae*, and *Tannerellaceae* (Figure 5A). At the genus level, the abundances of *ermB*, *aph*(*3*”)-*Ib*, and *aadA5* were strongly correlated with *Escherichia*, *Streptococcus*, *Enterococcus_I*, *Neisseria*, and *Acinetobacter* (Spearman’s ρ > 0.7, *p* < 0.05), while *aph*(*3*”)*-Ib*, *aadA5*, and *bla*_OXA‑129_ were strongly negatively correlated with *Staphylococcus* and *Clostridium_J* (Figure 5B). Only *aph*(3”)-*Ib* was strongly correlated with *Clostridium_Q* *.*

**Figure 5:**
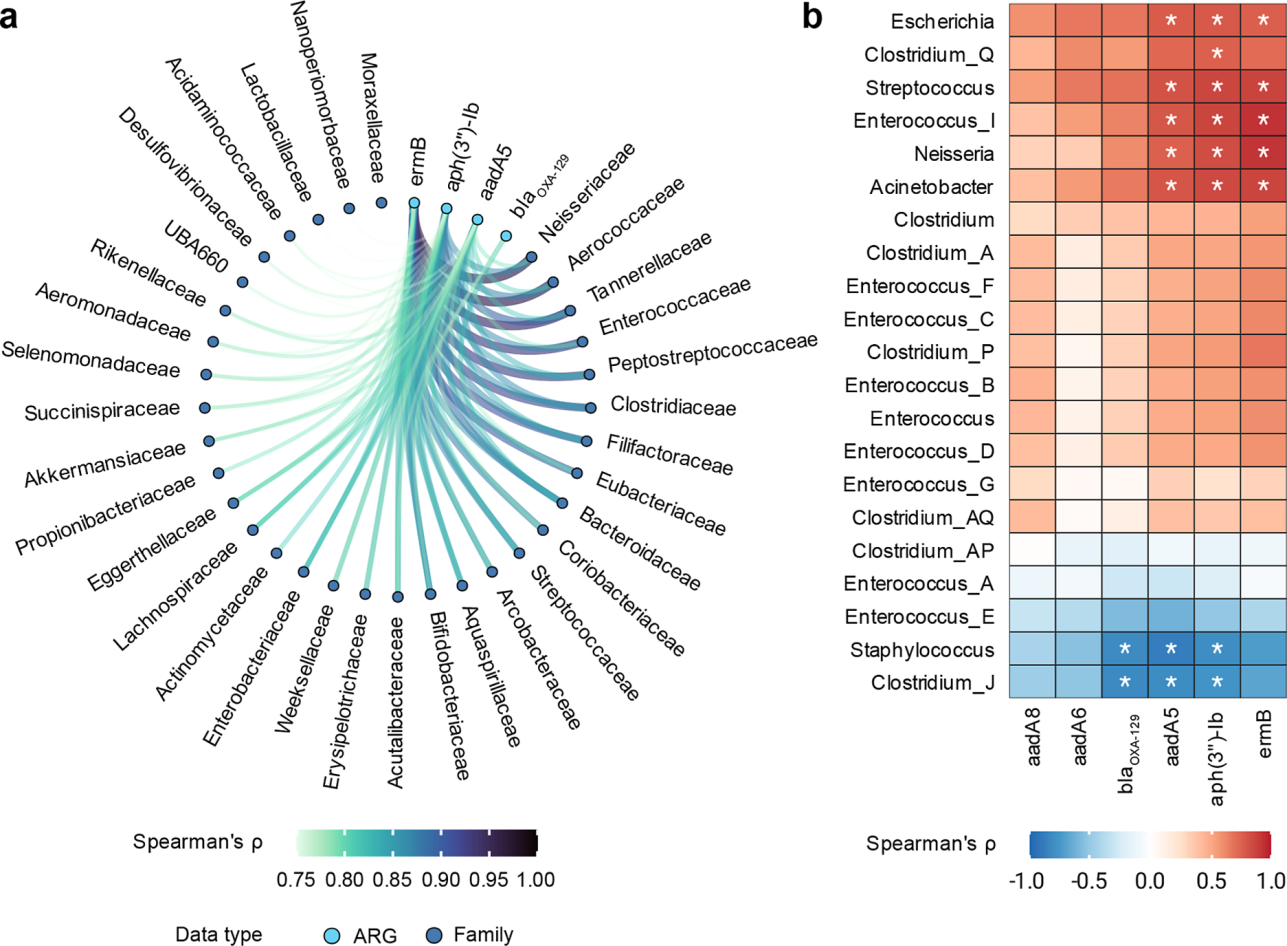
Cross-association analysis between selected Risk Level I ARG and bacterial taxon abundances at family and genus levels. (A) Network plot of positive correlations between selected Risk Level 1 ARGs and bacterial families. Only correlations with Spearman’s *p* > 0.75 are shown for visual clarity. (A) circular bipartite layout was used for the network. Nodes are ARGs (cyan blue) and bacterial taxa (medium blue) and are arranged as points along a circle, while edges are correlations between nodes and are drawn as lines. Correlation strength is displayed by color hue, width, and line opacity. (B) Heatmap of positive and negative correlations between selected Risk Level 1 ARGs and bacterial genera including known human pathogens. Positive correlations (Spearman’s ρ > 0) are shown in red, and negative correlations (Spearman’s ρ < 0) in blue. Hierarchical clustering of ARGs (columns) and genera (rows) was performed separately using a distance matrix (1–Spearman’s p) with unweighted pair group method with arithmetic mean (UPGMA) agglomeration. *, *p* < 0.05.

### 3.7 Antibiotic Residues Shaping the Bacteria Community, Resistome and MGEs

Redundancy analysis (RDA) was conducted to assess the role of antibiotic residues in shaping the composition of the bacterial community, resistome (ARGs), and mobilome (MGEs) in influent, effluent, and sludge samples from three WWTPs (Supporting Information Figure S8). The following antibiotic residue concentrations demonstrated multicollinearity and were removed from the analysis: ciprofloxacin, norfloxacin, levofloxacin, ofloxacin (fluoroquinolones), azithromycin (macrolide), and rifamixin (rifamycin). For the bacterial community, two RDA axes explained 63.1% of the total variation. Influent samples were strongly associated with acetyl sulfamethoxazole (ANOVA-like permutation test, *p* < 0.01), clarithromycin (*p* < 0.01), and sulfamethoxazole (*p* < 0.05), sludge samples with miconazole (*p* < 0.01) and doxycycline (*p* < 0.05), and effluent samples with trimethoprim (*p* < 0.01). In the resistome, two axes explained 36.4% of the total variation, with acetyl sulfamethoxazole (*p* < 0.01) associated with influent samples and miconazole (*p* < 0.05) with the sludge samples. For MGEs, two axes explained 40.8% of the total variation, with acetyl sulfamethoxazole (*p* < 0.01) linked to influent samples and miconazole (*p* < 0.05) to sludge. No other antibiotics showed significant associations (*p* ≥ 0.05), and the strongest associations corresponded to compounds with high concentrations in influents (e.g., sulfamethoxazole, clarithromycin), indicating that RDA reflects exposure patterns rather than revealing new causal relationships.

## 4 Discussion

Metagenomic sequencing revealed the occurrence and fate of a broad spectrum of ARGs and MGEs in the effluents of three WWTPs in Barcelona subjected to different water reuse treatments, as well as in their anaerobically digested sludge, which is applied to agricultural soil as an organic fertilizer. Detailed bioinformatics analyses delineated the responses of ARG target drug classes, high-risk ARGs, and MGEs to advanced treatment processes. Consistent with previous studies, the relative abundance of total ARGs decreased across all three WWTPs.^14,17,54^ Additionally, the total abundance of ARGs, expressed as ARGs per cell, was similar among the three WWTP effluents studied, and consistently fell into level 4 of the AMR exposure ranking scheme (<0.5 ARGs/cell) proposed by Yin et al.^54^ Within this framework, lower levels indicate reduced potential exposure to ARGs through environmental pathways, whereas higher levels reflect greater public and environmental health risks. The classification of our tested effluents within the level 4 rank is notably lower than the majority of the 468 effluents tested globally in their study, where most fell into higher exposure categories.^54^ In contrast, the relative abundances of MGEs in our effluents were comparable to those reported for many effluents in the global data set, suggesting that, despite lower ARG loads in the effluents, their mobilization potential is still of-concern.^55^ These findings are particularly relevant for reclaimed water reuse, as they indicate a comparatively low ARG exposure risk but highlight the need for continued monitoring of MGEs that may facilitate ARG dissemination.

The three studied influents originate from similar urban sources, and as such, they exhibited comparable profiles of ARGs, MGEs, and bacterial community composition. Despite differences in advanced tertiary treatments, a consistent pattern emerged in which certain ARG classes were more effectively removed from effluents than others. These included genes conferring resistance to diaminopyrimidines, phosphonic acids, nucleosides, nitroimidazoles, and MLS, which are often associated with planktonic bacteria and MGEs with lower transfer efficiency, making them more susceptible to physical and chemical removal processes in both conventional and advanced wastewater treatments.^55–59^ In contrast, ARGs conferring resistance to aminoglycosides, tetracyclines, fluoroquinolones, sulfonamides, glycopeptides, and beta-lactams were less effectively removed, and are more often associated with biofilm-forming bacteria and highly transferable MGEs, which are less susceptible to removal.^55,57–61^ Importantly, most antibiotics that exceeded their PNECs^47^ in our study corresponded to these recalcitrant drug classes. For instance, ciprofloxacin (quinolone, PNEC: 64 ng/L) exceeded its threshold in all influent, effluent, and sludge samples, while ofloxacin (quinolone, PNEC: 500 ng/L) surpassed its limit in influent and sludge samples but not in effluents. Doxycycline (tetracycline, PNEC: 100 ng/L) exceeded its threshold in 67% of influent samples, and sulfamethoxazole (sulfonamide, PNEC: 600 ng/L) was consistently above its limit in 100% of influent samples. The persistence of these compounds above selective concentrations likely contributes to the limited removal, or even enrichment, of genes conferring resistance to these antibiotic classes in the effluents. These observations are also consistent with well-established differences in antibiotic stability in wastewater systems. Relatively persistent compounds, such as fluoroquinolones, macrolides, and sulfonamides, are known to resist biological degradation and sorb to solids, favoring their persistence in effluents and sludge and sustaining selective pressure for resistance, whereas less stable antibiotics are more rapidly attenuated during treatment, which may partly explain the compound-specific removal efficiencies observed.^62^

The resistome and mobilome composition of G-V effluent was distinct from P.LL and F.LL (Figure S3A,B), whereas total abundance did not mark G-V as an outlier (Figure S7). This points out the importance of examining changes in relative abundance at multiple levels, including the overall resistome, target drug classes, and ARG alleles rather than relying solely on total abundance.^63^ G-V effluents were enriched in genes predicted to encode multidrug efflux pumps and disinfectant tolerance. Multidrug efflux pump genes are often chromosomally encoded and their products have physiological roles other than antibiotic resistance, including the excretion of nonantibiotic toxic compounds and cellular metabolites outside the cell.^64^ This broad metabolic function makes them poor monitoring targets for both antibiotic resistance and horizontal gene transfer risk due to their ubiquity in bacterial genomes and widespread presence in different environments.^63^ Consistently, the G-V WWTP was particularly effective eliminating highly transferable MGEs (Figure 4B), such as IS26, plasmids, ISCR1, *intI*2, *intI*3, ISCR5, and ISCR5b, that play a major role in the dissemination of ARGs between Gram-negative bacteria, including clinically relevant pathogens.^15,50–53,65^ Focusing on high-risk (Rank I) ARGs^48^ reinforces this pattern, as G-V achieved the most efficient removal of Rank I genes (Figure 3) in parallel with a greater reduction of MGE markers (Figure 4B). These results indicate that limiting gene-transfer pathways, rather than merely lowering total ARG abundance, is critical to reducing clinically relevant resistance in effluents.

The differences observed between WWTP effluents may be explained by variations in their bacterial community composition (Figures 1 and S4). In particular, the microbiome of the G-V WWTP effluent was different from those in P.LL and F.LL effluents, which were similar. These differences were likely linked to the distinct configuration of the G-V tertiary treatment, uniquely combining a biological step (membrane bioreactor) with a physical separation (ultrafiltration) before UV disinfection and chlorination. The G-V effluent is characterized by a microbial community enriched in *Pseudomonadota* (particularly *Rhodobacteraceae*), *Nitrospirota*, and *Deinococcota*. *Rhodobacteraceae* include versatile denitrifiers, *Nitrospirota* are key nitrite oxidizers, and *Deinococcota* is well-known for their high resistance to oxidative stress and DNA-damage.^59,66–69^ In turn, the P.LL and F.LL plants, which rely only on physical separation processes before the final disinfection, yielded effluents enriched in *Bacteroidaceae* and *Tannerellaceae* (*Bacteriodota*), and retained taxa of the families *Streptococcaceae* and *Lachnospiraceae* (*Bacillota*). Both groups are recognized as frequent carriers of MGEs and ARGs and are strongly associated with human feces.^70–72^ Taken together, the G-V tertiary configuration (MBR + ultrafiltration + UV/chlorination) enriched nutrient-removal and oxidative-stress–tolerant taxa while reducing the abundance of fecal-associated taxa and transferable MGEs associated with clinically relevant ARGs. P.LL and F.LL plants (physical separation + UV/chlorination), in turn, favor the persistence of taxa linked to human excreta. These results are consistent with previous studies showing that ultrafiltration and advanced membrane bioreactor processes are more effective than conventional physical separation systems in reducing resistant bacteria and human fecal–associated taxa.^73–76^

## 5 Selecting Sentinel ARGs for Monitoring in Reclaimed Wastewater and Sludge

A critical need in wastewater surveillance is to identify ARGs that reliably indicate anthropogenic inputs. Because many ARGs are ubiquitous in soils and aquatic systems, monitoring should prioritize high-risk targets strongly linked to fecal bacteria, mobility, and clinically relevant pathogens. Accordingly, sentinel *ARGs* should (i) reflect anthropogenic prevalence, (ii) be associated with MGEs and pathogenic hosts (Rank I ARGs), (iii) remain resilient to the advanced treatments used for reclaimed water, and (iv) be prevalent/abundant in the final effluent.

In our study, several Rank I ARGs persisted in reclaimed effluents and sludge regardless of treatment configuration, including genes predicted to confer resistance to aminoglycosides (*aadA6*, *aadA5*, *aadA8*, and *aph*(*3*’’)*-Ib*) and macrolides (*ermB* and *mphA*), and the beta-lactamase-encoding gene *bla*_OXA‑129_. Their consistent detection across effluents highlights their value as sentinel markers for monitoring the dissemination of antibiotic resistance in reclaimed water. In addition, genes particularly enriched in sludge samples, such as *sul3*, may represent suitable candidates for inclusion in monitoring frameworks targeting soils impacted by treated sludge application. Compared with conventional WWTPs focusing on secondary treatment, reuse-oriented systems showed distinct antibiotic resistance profiles characterized by lower overall ARG exposure levels but a persistence of clinically relevant, mobile resistance determinant.^77,78^ Prior indicator-gene analyses of influent and secondary effluents have also prioritized aminoglycoside- and macrolide-resistance determinants as high-concern targets in wastewater.^79,80^ Tarek and Garner identified *aadA*/*aph* and mph families among top signals, while Bengtsson-Palme et al. likewise emphasized *ermB* together with sulfonamide/aminoglycoside markers.^47,81^ Our reclaimed-water panel converges at the mechanism/gene-family level (*aadA*/*aph*, *ermB*, *mph*), but the alleles differ (e.g., *mphA* and *aadA*5/6/8 here versus other *mph*/*aadA* variants in global wastewater data sets). These differences likely reflect matrix and selection context (tertiary reclaimed effluents and digested sludge versus predominantly secondary sewage), geography, and database/version effects. Together, this suggests that family level sentinels are robust across studies, whereas allele-level choices should be locally validated for reclaimed-water monitoring. Given the variability of alleles detected across sites and data sets, we propose that sentinel markers should preferentially be selected at the gene-family level. In our study, this includes *bla*_OXA_ (β-lactamases), *aadA* and *aph* (aminoglycoside-modifying enzymes), *ermB* and *mph* (macrolide-resistance genes), as well as *sul* (sulfonamide-resistance genes). Focusing surveillance on these families rather than on individual alleles would provide more robust indicators across different treatment configurations and geographical contexts. This strategy would further benefit from the use of sufficiently degenerate primers to ensure coverage of the allelic diversity within each family when using amplification-based surveillance methods, while still capturing their overall role as indicators of anthropogenic antibiotic resistance.^82^

The cross-association analysis provides additional support for the selection of aminoglycoside- and macrolide-resistance genes as sentinel markers for reclaimed wastewater and sludge. The strong correlations observed between key Rank I ARGs (e.g., *ermB*, *aadA5*, *aph*(3″)*-Ib*) and bacterial taxa associated with human pathogens, including *Enterobacteriaceae*, *Streptococcaceae*, *Enterococcaceae* and *Neisseriaceae*, indicate that these genes remain linked to clinically relevant hosts after advanced wastewater treatment.^82–84^ At the same time, their associations with families comprising human commensals and opportunistic pathogens (e.g., *Bacteroidaceae*, *Tannerellaceae*, *Lachnospiraceae* and *Bifidobacteriaceae*) highlight the role of the gut microbiota as a reservoir facilitating persistence and potential dissemination of resistance.^85–88^ The detection of consistent genus-level correlations with well-known pathogenic genera such as *Escherichia*, *Streptococcus*, *Neisseria*, *Acinetobacter*, and *Enterococcus* further reinforces the epidemiological relevance of these ARGs.^80,89,90^ Together, these patterns suggest that the selected Rank I ARGs capture both pathogenic risk and ecological connectivity, supporting their suitability as sentinel targets for monitoring antimicrobial resistance in reclaimed water and treated sludge.

Metagenomic analysis is particularly powerful for the initial identification of such candidate genes, as it allows a comprehensive and unbiased assessment of the resistome. However, because ARG profiles vary between WWTPs, treatments, and regions, site-specific studies are essential to determine the most relevant monitoring targets. Once candidate genes are identified, efforts should shift toward optimizing resources by focusing on their families, enabling routine surveillance that is both cost-effective and informative. This stepwise approach, from metagenomic discovery to family level target assays, provides a practical route to operationalize the AMR monitoring requirements of the EU agricultural water reuse regulation. Incorporating these sentinel ARG families into monitoring programs would not only improve our ability to track wastewater-derived resistance but also provide an evidence base for regulatory frameworks on wastewater and treated sludge reuse. Given the increasing importance of reclaimed water for irrigation and soil fertilization, selecting and standardizing appropriate monitoring targets is crucial to balance sustainability goals with public health protection.

## Supplementary Material





## Notes

Details on detected compounds in influent, effluent, and sludge samples across wastewater treatment plants; top 10 most increased and decreased bacterial phyla (upper panels) and families (lower panels); principal component analysis of ARGs, MGEs, and bacterial communities; bacterial community composition at phylum and family levels; alpha diversity metrics (Chao1 richness and Simpson’s evenness) for ARGs and MGEs; total abundances of ARGs and MGEs by sample type and treatment plant; redundancy analysis linking microbial features with antibiotic residues; analytical parameters, surrogate characteristics, limits of detection and quantification for antibiotics in water and sludge; concentrations of antibiotics in influent, effluent, and sludge samples; software versions, databases, and bioinformatics pipelines used; list of antibiotic resistance genes and associated risk rankings; marker genes used for prokaryotic cell abundance estimation (PDF)
List of antibiotic resistance genes (ARGs) with identical amino acid sequences in CARD (v3.2.9) and SARG (v2023-04-21) databases, along with their assigned SARG risk levels (XLSX)
