# Supplementary material for: Metagenomic Assessment of Full-Scale Wastewater Treatment Plants Identifies Sentinel Antibiotic Resistance Gene Families for Monitoring Reclaimed Wastewater and Treated Sludge
Source: Environ Sci Technol. 2026 Feb 4;60(6):4632–47. doi: 10.1021/acs.est.5c13541 (PMC12918712; doi:10.1021/acs.est.5c13541)
Supplement: Supplementary file 1 [file es5c13541_si_001.pdf]

Supplementary Information

Metagenomic assessment of full-scale wastewater treatment plants identifies sentinel antibiotic resistance gene families for monitoring reclaimed wastewater and treated sludge.

Brown, Liam P.[a], Marizzi, Anna[b]., Borrego, Carles M.[c,d], Giulia, Gionchetta[b], Zhao, Zhengzheng[e]., Carneiro, Rodrigo B[f]., Gago-Ferrero, Pablo[b], Matamoros, Victor[b], Subirats, Jessica[b]

[a]Ottawa Laboratory (Carling), Canadian Food Inspection Agency, 960 Carling Ave., bldg.22, Ottawa, ON K1A 0C6, Canada.

[b]Department of Environmental Chemistry, Institute of Environmental Assessment and Water Research (IDAEA), Severo Ochoa Excellence Center, Spanish Council of Scientific Research (CSIC), Jordi Girona 18-26, Barcelona, E-08034, Spain.

[c] Catalan Institute for Water Research (ICRA-CERCA), Emili Grahit 101, Girona E-17003, Spain.

[d]Grup d'Ecologia Microbiana Molecular, Institut d'Ecologia Aquàtica, Universitat de Girona, Campus de Montilivi, Girona E-17003, Spain.

[e]National Key Laboratory of Agricultural microbiology, College of Life Science and Technology, National Engineering Research Center of Microbial Pesticides, Huazhong Agricultural University Wuhan 430070, PR China.

[f]Laboratory of Chromatography (CROMA), São Carlos Institute of Chemistry, University of São Paulo (USP), 400, Trabalhador São-Carlense Ave., São Paulo, São Carlos, 13566-590, Brazil.

*Corresponding author: Jessica Subirats

Department of Environmental Chemistry, IDAEA-CSIC, Jordi Girona, 18-26, 08034, Barcelona, Spain.

Email: jessica.subirats@cid.csic.es

Supplementary figures

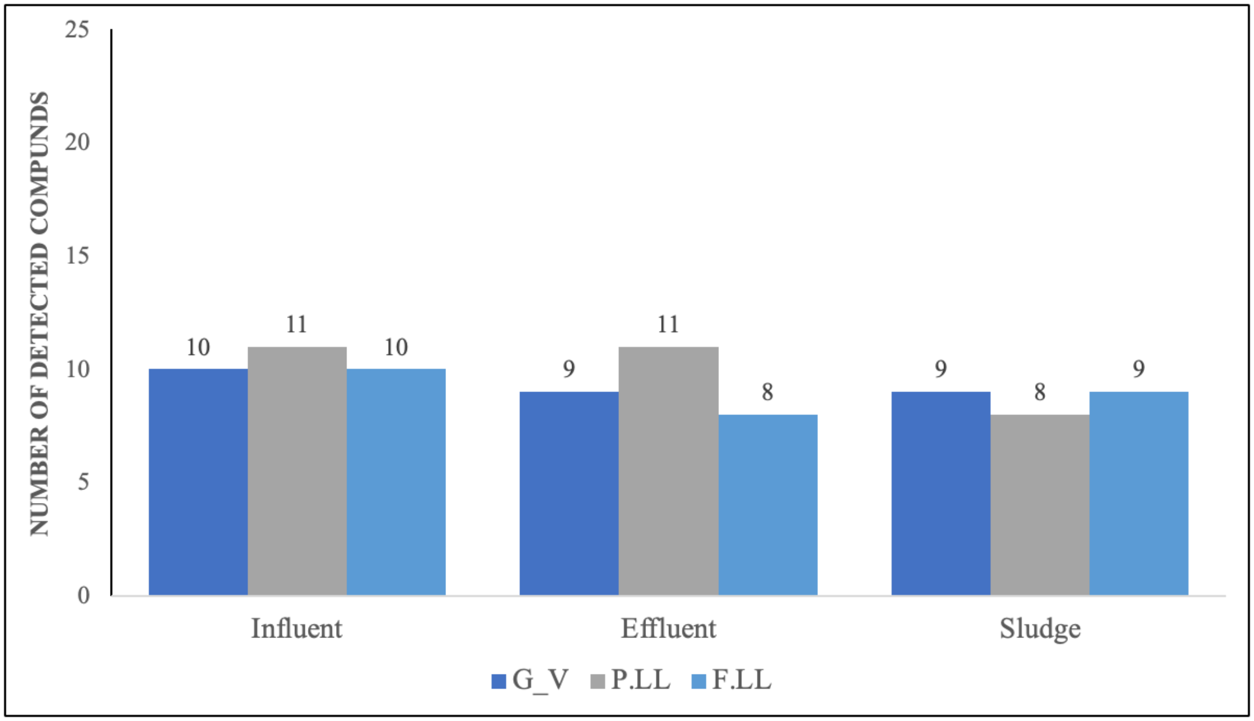

Figure S1: Number of detected compounds in influent, effluent, and sludge samples from the Prat de Llobregat (P.LL), Sant Feliu de Llobregat (F.LL) and Gavà-Viladecans (G-V) WWTPs, out of the 25 compounds screened.

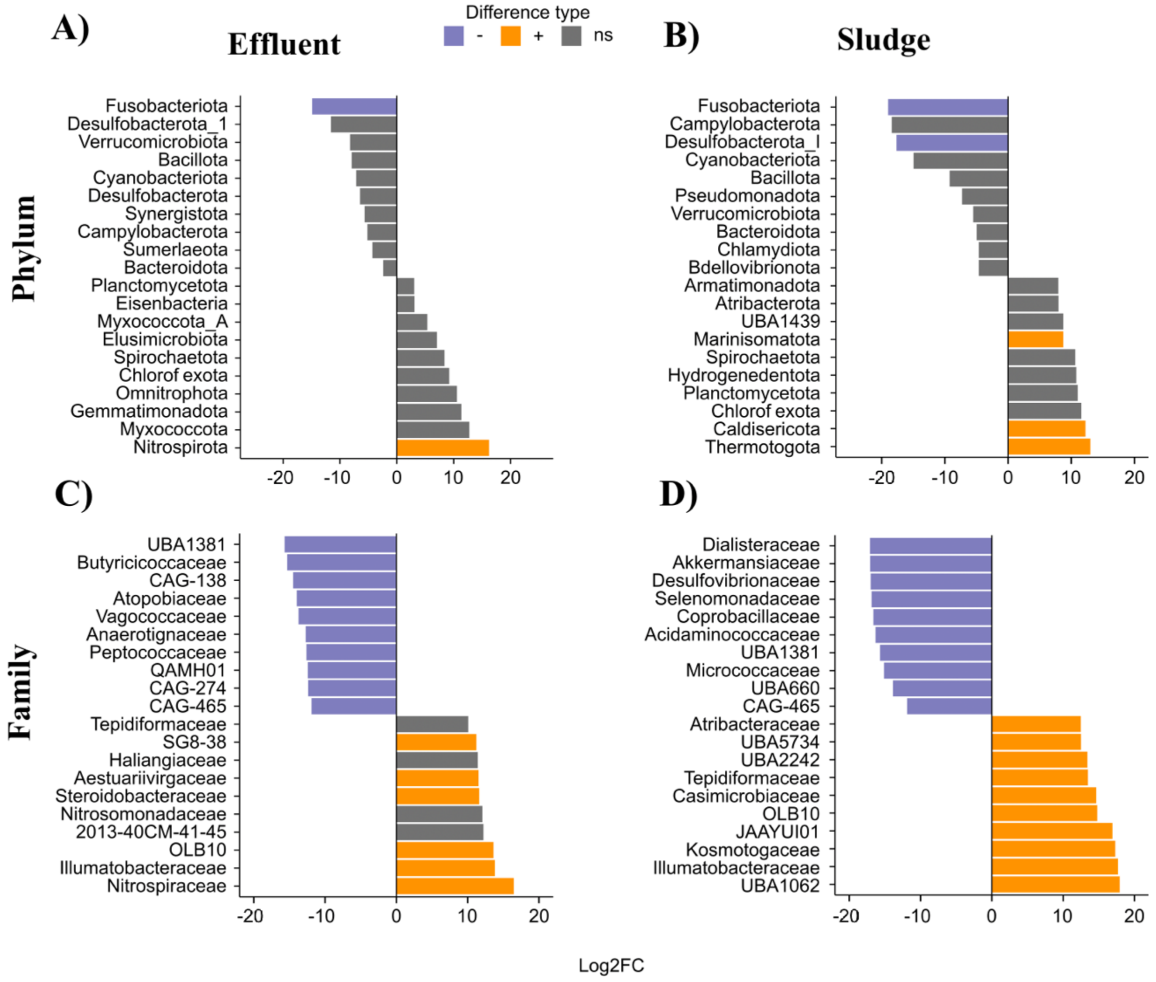

Figure S2: Top 10 most increased and decreased bacterial phyla (upper panels) and families (lower panels). Fold-changes in relative abundance are shown on the x-axis in Log2 scale (Log2FC) for effluent (A, C) and sludge (B, D) samples. Positive Log2FC values indicate enrichment, while negative values indicate removal. For reference, a Log2FC of 1 corresponds to a 2-fold increase (doubling), a Log2FC of 2 to a 4-fold increase (quadrupling), and a Log2FC of −1 to a 2-fold decrease (halving). Analyses were performed by pooling samples across the three WWTPs (n = 9 per sample type: 3 plants × 3 replicates). Taxa were considered significantly increased or decreased if $|Log2FC_{std}| > 10$ and $p < 0.05$. Purple, significantly decreased; orange, significantly increased; grey, not significant (ns).

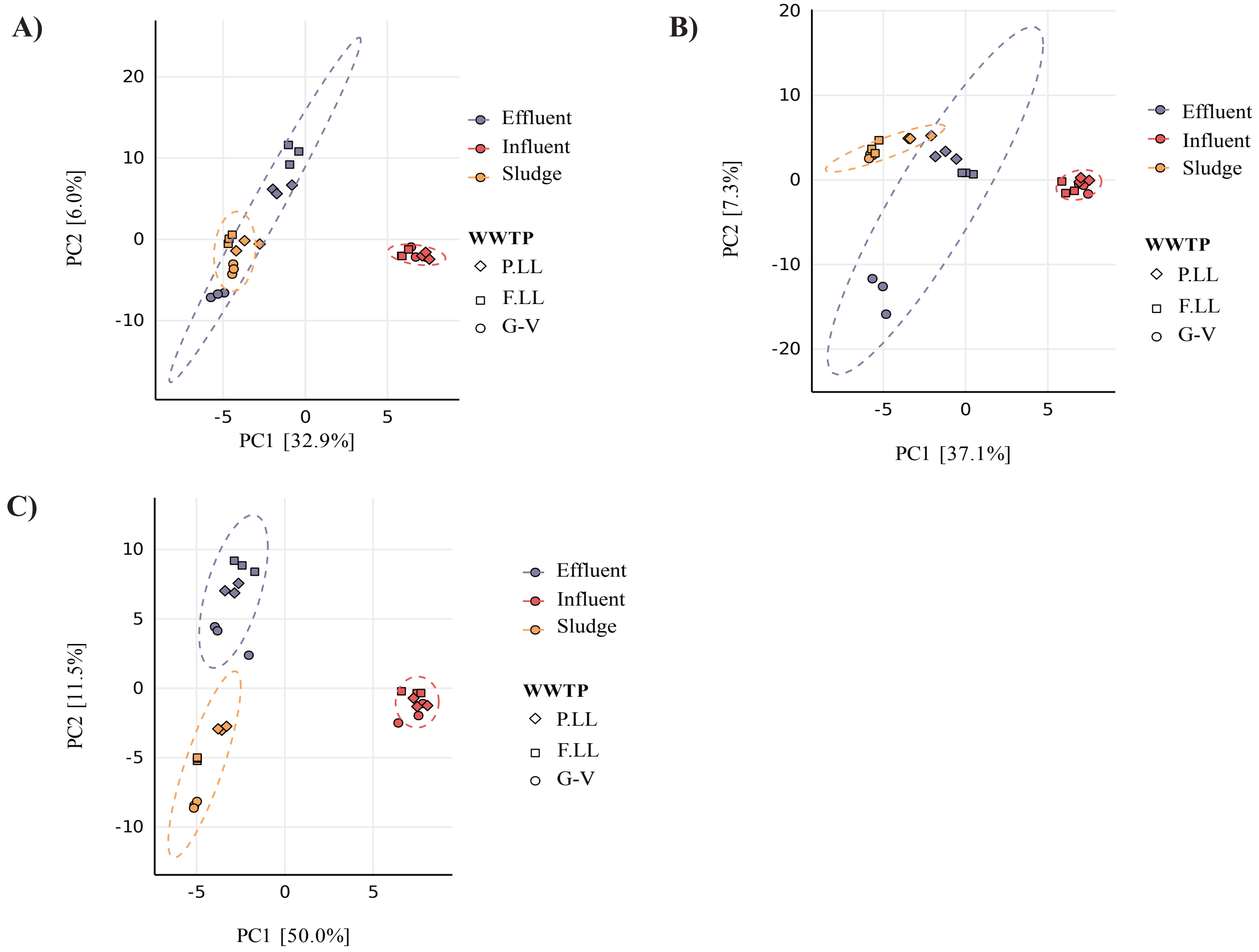

Figure S3: Principal component analysis (PCA) ordination plots of center log ratio-transformed relative abundances of ARGs (A), MGEs (B), and bacterial taxa (C). Samples are coloured by sample type and shaped by WWTP. Percentages of total variation explained by each axis are displayed within square brackets in axis titles. Dashed ellipses correspond to 95% confidence intervals for each sample type. P.LL, Prat de Llobregat; F.LL, Sant Feliu de Llobregat; G-V, Gavà-Viladecans.

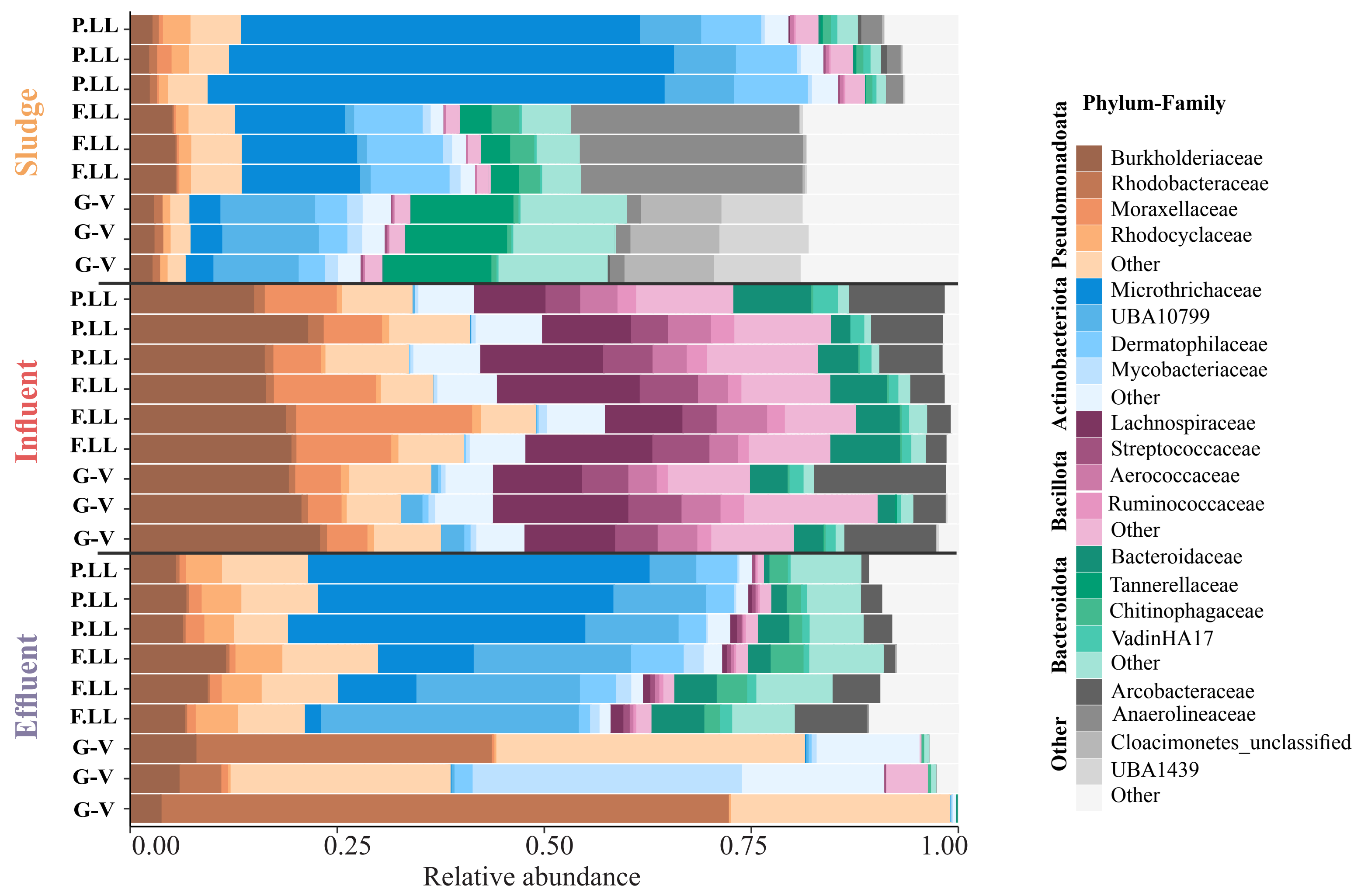

Figure S4: Proportion of bacterial communities in the 27 metagenomes evaluated at the phylum and family levels. The top 5 most prevalent bacterial families are represented by different shades of colours, and the term "Other" represents the bacterial communities present in a smaller abundance.

A) Chao1_ARGs

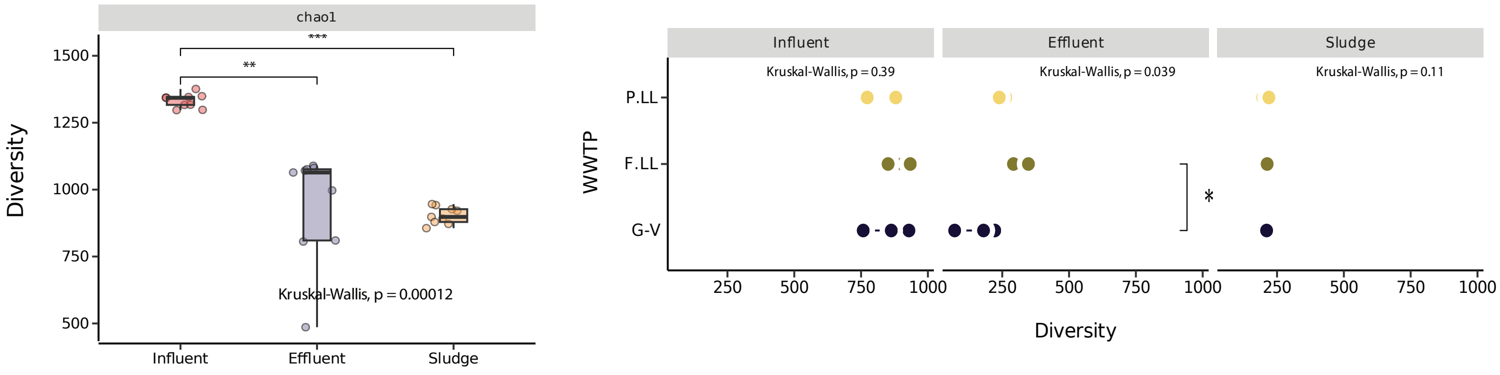

B) Chao1_MGEs

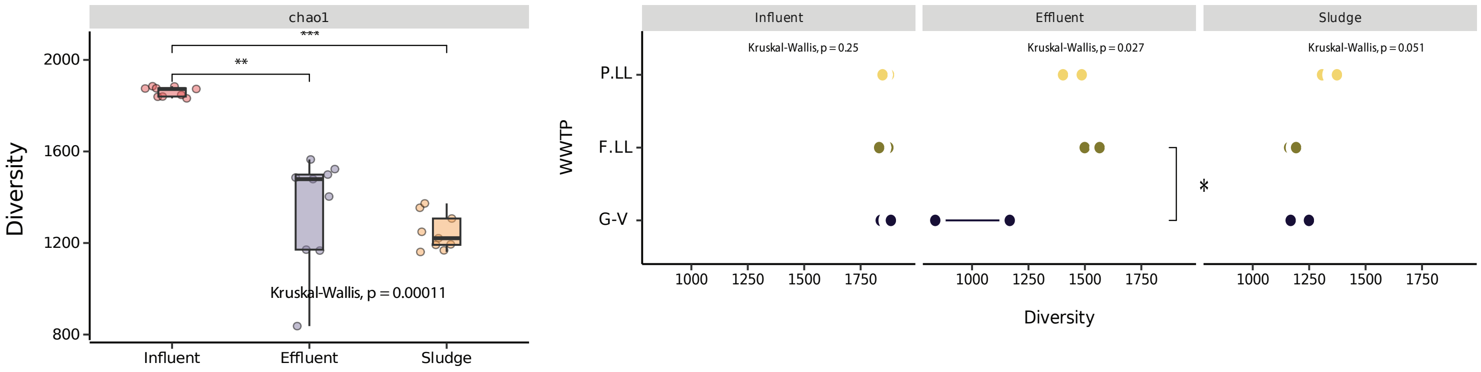

Figure S5: Alpha diversity measured by the Chao1 richness estimator for (A) ARGs and (B) MGEs, grouped by sample type (left panels) and by WWTP (right panels). Dunn's test with Bonferroni correction was used for post-hoc pairwise comparisons. *, p <0.05; **, p < 0.01; ***, p < 0.001; ****, p < 0.0001.

A) Simpson's evenness_ARGs

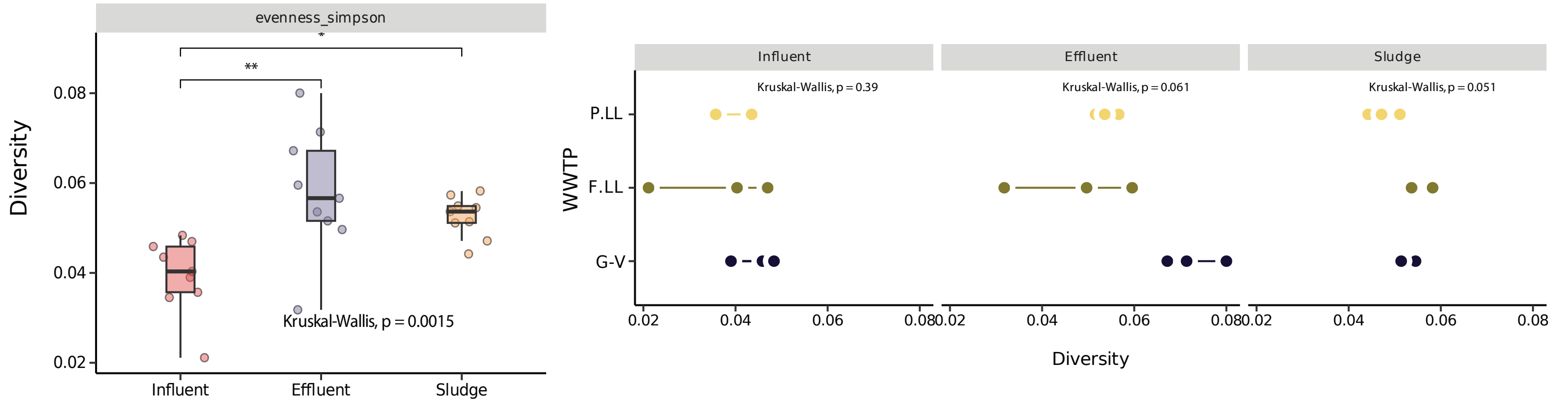

B) Simpson's evenness_MGEs

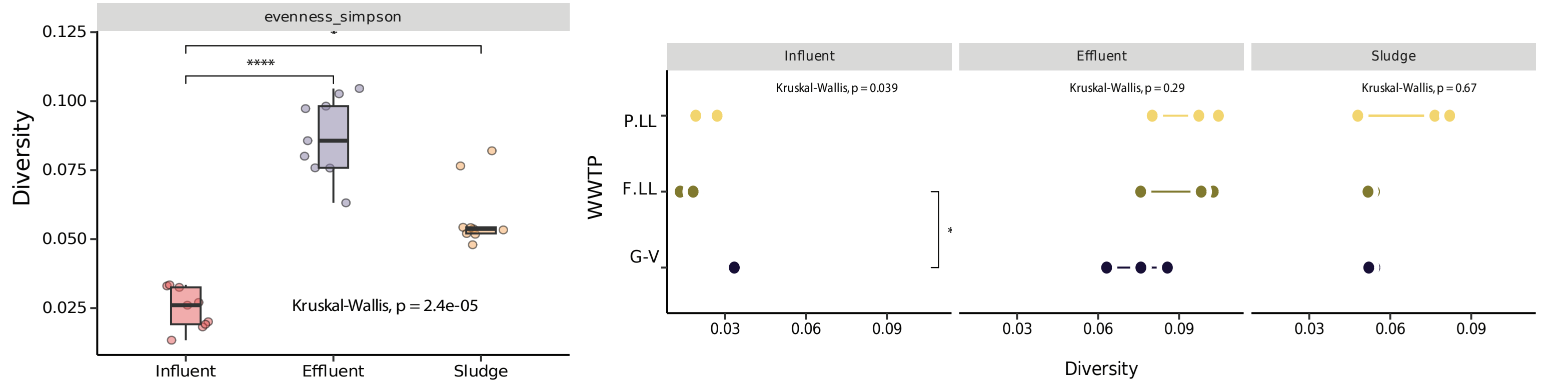

Figure S6: Alpha diversity measured by Simpson's evenness index for (A) ARGs and (B) MGEs, grouped by sample type (left panels) and by WWTP (right panels). Dunn's test with Bonferroni correction was used for post-hoc pairwise comparisons. *, p <= 0.05; **, p <= 0.01; ***, p<= 0.001; ****, p <= 0.0001.

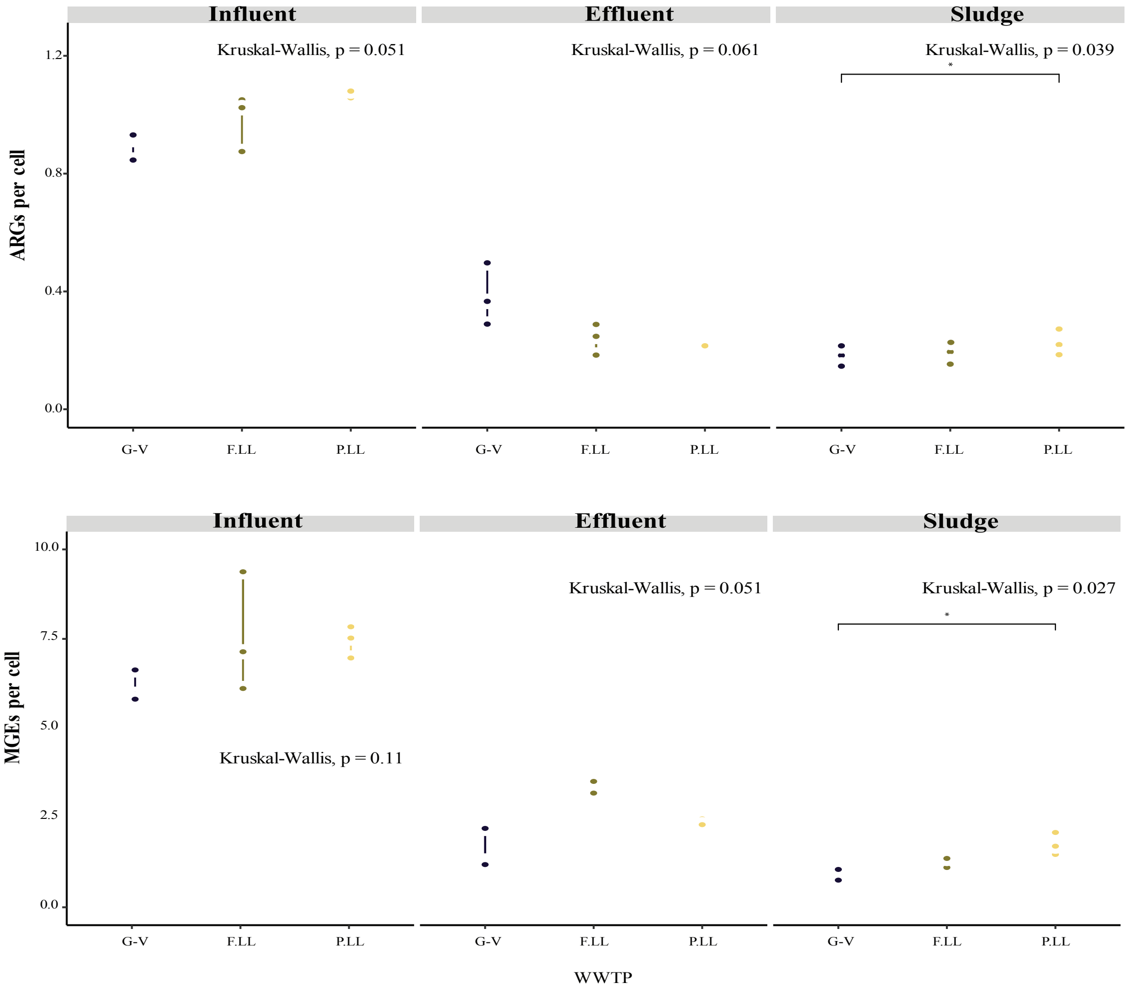

Figure S7: Total ARG (upper panel) and MGE (lower panel) abundances in influent, effluent, and sludge samples from the Prat de Llobregat (P.LL), Sant Feliu de Llobregat (F.LL), and Gavà-Viladecans (G-V) WWTPs. Dunn's test with Bonferroni correction was used for post-hoc pairwise comparisons. *, $p < 0.05$; **, $p < 0.01$; ***, $p < 0.001$; ****, $p < 0.0001$. Target detection thresholds of prop cov >= 0.7 and fold cov >= 0.7 were used to determine if an ARG or MGE was present or absent.

RDA (CLR) of bacterial taxa

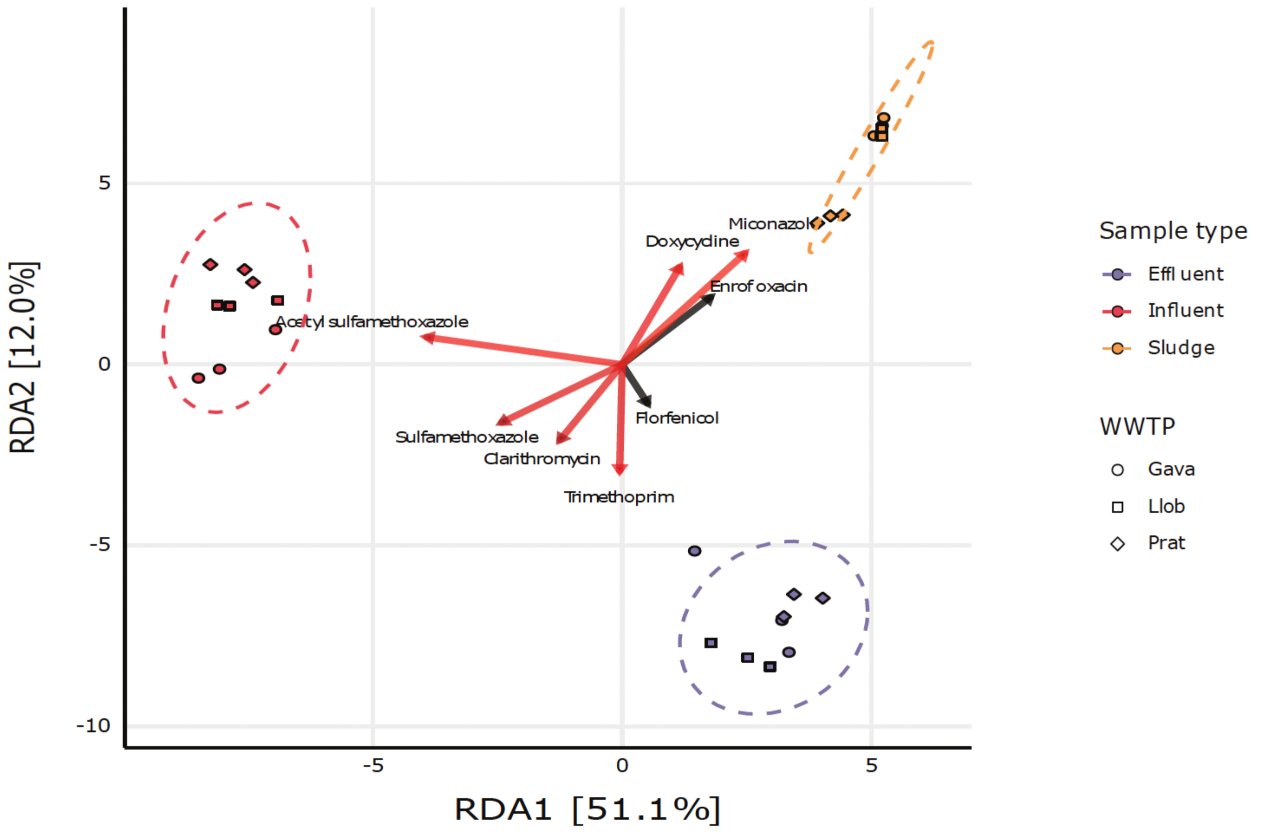

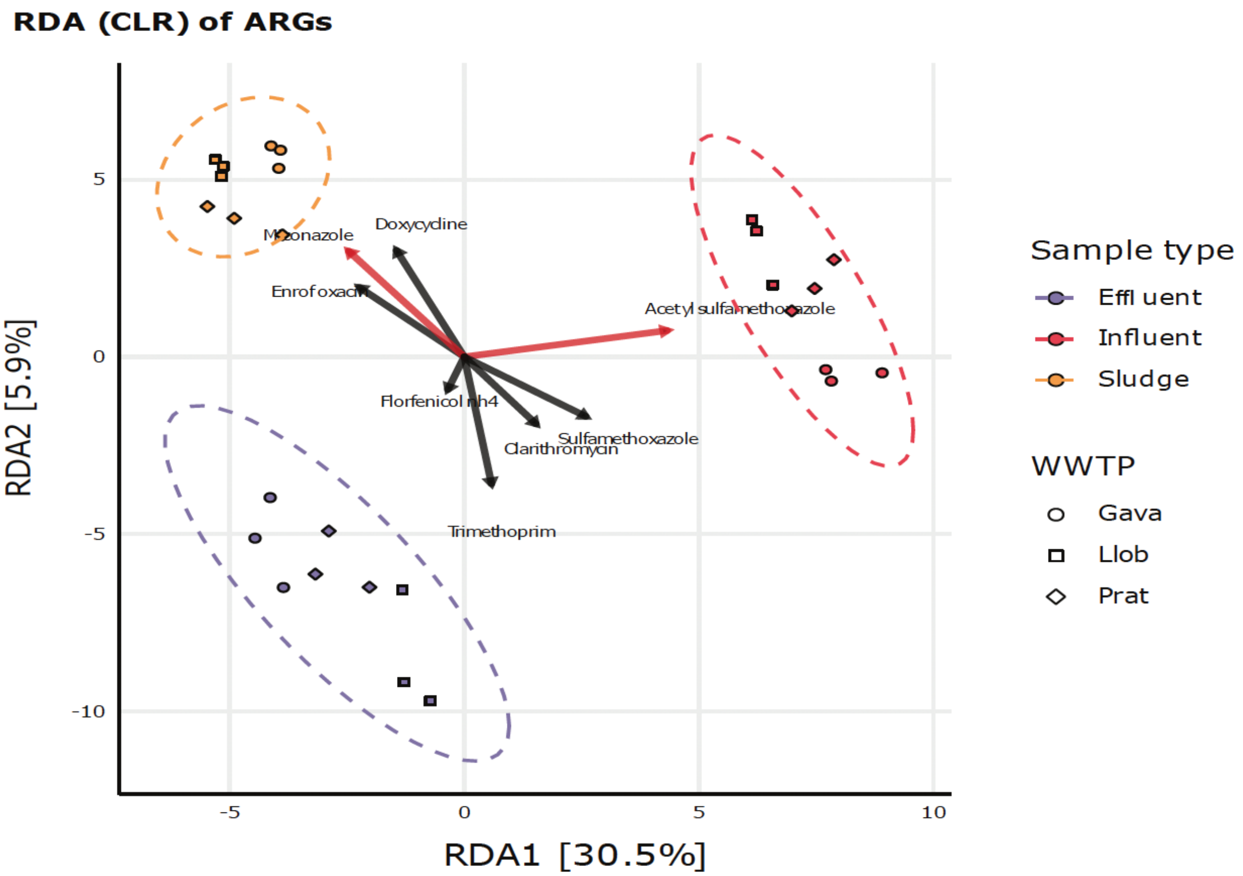
RDA (CLR) of ARGs
Doxycycline
Acetyl sulfamethoxazole
Florfenicol nh4
Sulfamethoxazole
Clarithromycin
Trimethoprim
5
0
-5
-10
-5
0
5
10
RDA2 [5.9%]
RDA1 [30.5%]
Sample type
Effluent
Influent
Sludge
WWTP
Gava
Llob
Prat

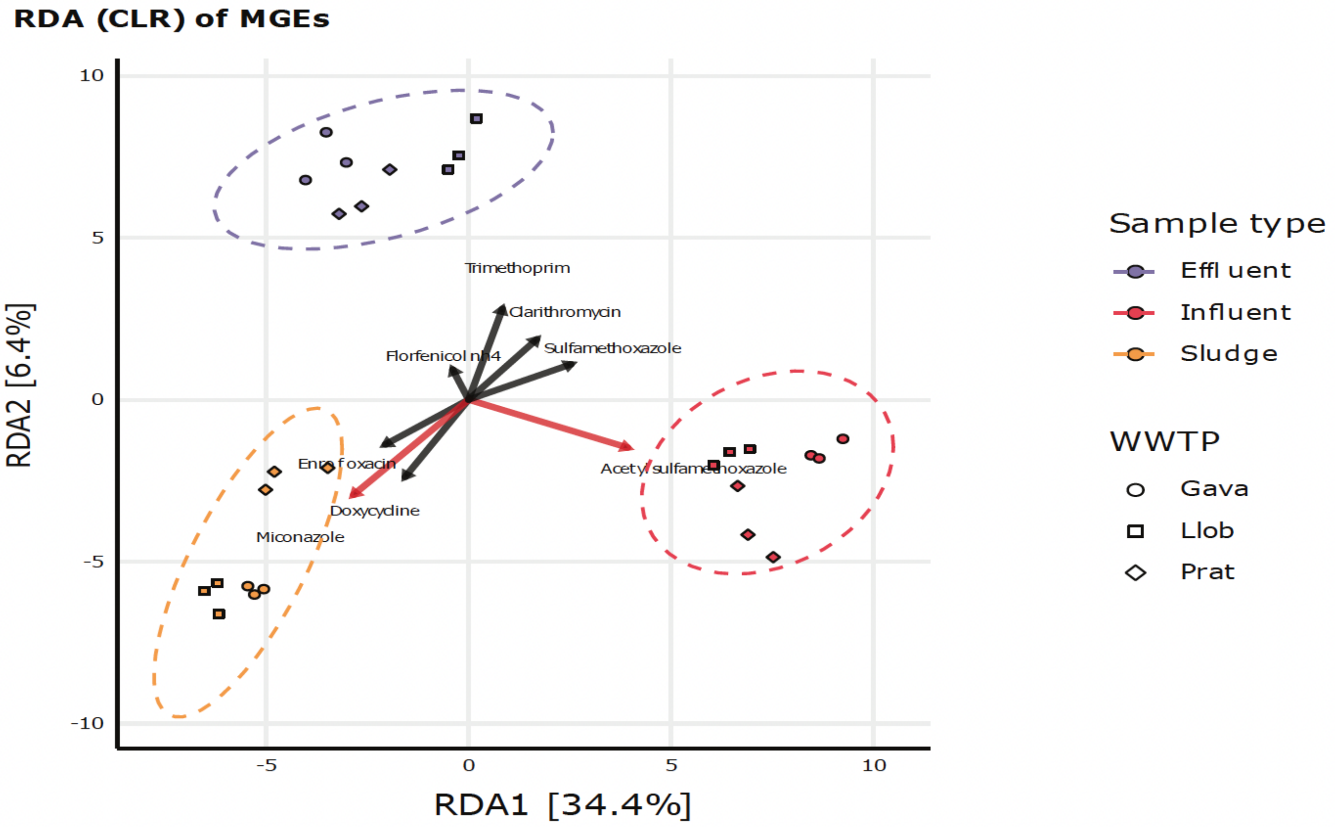

FigureS8: Redundancy analysis (RDA) biplot of center log ratio-transformed bacterial taxa, ARG, or MGE abundances, with antibiotic residue concentrations as constraining variables and WWTP identity as a conditioning variable. Red arrows indicate variables with statistically significant associations (ANOVA-like permutation test, $p < 0.05$).

Supplementary tables

Table S1: Characteristics of isotopically labeled surrogates used for quantification of antibiotics in water and sludge matrices. RT, retention time; m/z, mass-to-charge ratio; LOQ; limit of quantification; LOD: Limit of detection.

					Water [ng/L]		Sludge [ng/g]	
**Surrogate**	**Formula**	**RT**	**m/z**	**R^2**	**LOQ**	**LOD**	**LOQ**	**LOD**
**Chlortetracycline**	C22H23ClN2O8	5.7	479.12157	0.97	25	7.5	5	1.5
**Ciprofloxacin-d8**	C17H10D8FN3O3	4.63	340.19071	0.93	12.5	3.75	2.5	0.75
**Clindamycin-d3**	C18H30D3ClN2O5S	6.83	428.20598	1.00	1.25	0.37	0.25	0.07
**Enrofloxacin-d5**	C19H17D5FN3O3	4.7	365.20318	0.98	12.5	3.75	2.5	0.75
**Ofloxacin-d3**	C18D3H17FN3O4	4.45	365.16989	0.98	5	1.5	1	0.3
**Sulfamethizole-d4**	C9D4H6N4O2S2	4.49	275.0569	1.00	2.5	0.75	0.5	0.15
**Sulfamethoxazole D4**	C10D4H7N3O3S	4.75	258.0845	1.00	12.5	3.75	2.5	0.75
**Trimethoprim-d3**	C14H15D3N4O3	4.46	294.164	1.00	1.25	0.37	0.25	0.07

Table S2: Analytical parameters for selected antibiotics and antifungals in water and sludge matrices. Parameters include chemical formula, retention time (RT), mass-to-charge ratio (m/z), Chemical Abstracts Service (CAS) number, method linearity (R^2), limits of quantification (LOQ), and detection (LOD) for both water (ng/L) and sludge (ng/g).

							Water [ng/L]		Sludge [ng/g]	
**Family**	**Antibiotic**	**Formula**	**RT**	**m/z**	**CAS**	**R^2**	**LOQ**	**LOD**	**LOQ**	**LOD**
**Antifungals**	Miconazole	C18H14Cl4N2O	12.58	416.9905	(22916-47-8)	0.98	2.5	0.75	0.5	0.15
**Beta-lactams**	Cefalexin	C16H17N3O4S	4.44	348.10125	(15686-71-2)	1.00	25	7.5	5	1.5
**Diaminopyrimidines**	Trimethoprim	C14H18N4O3	4.31	291.14517	(738-70-5)	1.00	0.5	0.15	0.1	0.03
**Fluoroquinolones**	Ciprofloxacin	C17H18FN3O3	4.52	332.1405	(85721-33-1)	0.98	25	7.5	5	1.5
	Enrofloxacin	C19H22FN3O3	5.1	360.1718	(93106-60-6)	0.98	25	7.5	5	1.5
	Levofloxacin	C18H20FN3O4	4.58	362.15106	(100986-85-4)	0.99	2.5	0.75	0.5	0.15

	Ofloxacin	C18H20FN3O4	4.45	362.15106	(82419-36-1)	0.96	12.5	3.75	2.5	0.75
	Norfloxacin	C16H18FN3O3	4.5	320.1405	(70458-96-7)	0.99	50	15	10	3
**Lincosamides**	Clindamycin	C18H33ClN2O5S	6.62	425.18715	(18323-44-9)	1.00	0.5	0.15	0.1	0.03
	Lincomycin	C18H34N2O6S	4.38	407.22103	(154-21-2)	0.99	2.5	0.75	0.5	0.15
**Macrolides**	Azithromycin	C38H74N2O12^2+	5.63	375.26154	(83905-01-5)	0.97	2.5	0.75	0.5	0.15
	Clarithromycin	C38H69NO13	8.71	748.48417	(81103-11-9)	0.99	1.25	0.375	0.25	0.075
	Roxithromycin	C41H76N2O15	8.76	837.53185	(80214-83-1)	0.99	12.5	3.75	2.5	0.75
**Phenicols**	Florfenicol	C12H18Cl2FN2O4S^1+	4.87	375.03429	(73231-34-2)	1.00	5	1.5	1	0.3
**Rifamycins**	Rifaximin	C43H51N3O11	10.03	786.35964	(80621-81-4)	0.99	12.5	3.75	2.5	0.75
**Sulfonamides**	Acetyl-sulfamethoxazole	C12H13N3O4S	5.2	296.06995	(21312-10-7)	1.00	0.5	0.15	0.1	0.03
	Sulfacetamide	C8H10N2O3S	3.86	215.04849	(144-80-9)	1.00	5	1.5	1	0.3
	Sulfadiazine	C10H10N4O2S	4.09	251.05972	(68-35-9)	1.00	1.25	0.375	0.25	0.075
	Sulfaguanidine	C7H10N4O2S	3.04	215.05972	(57-67-0)	1.00	25	7.5	5	1.5
	Sulfamethizole	C9H10N4O2S2	4.49	271.03179	(144-82-1)	1.00	25	7.5	5	1.5
	Sulfamethoxazole	C10H11N3O3S	4.76	254.05939	(723-46-6)	1.00	1.25	0.375	0.25	0.075
	Sulfapyridine	C11H11N3O2S	4.28	250.06447	(144-83-2)	1.00	1.25	0.375	0.25	0.075
	Sulfathiazole	C9H9N3O2S2	4.13	256.0209	(72-14-0)	1.00	5	1.5	1	0.3
**Tetracyclines**	Doxycycline	C22H24N2O8	5.96	134.0715	(564-25-0)	1.00	0.5	0.15	0.1	0.03
	Oxytetracycline	C22H24N2O9	4.5	261.0973	(79-57-2)	0.99	2.5	0.75	0.5	0.15

Table S3: Versions of software packages, databases, and metadata files used for bioinformatics analysis, in order of their appearance in the main text.

Resource type	Name	Version	Environments	Parameters	Notes
Software	BaitCapture	v2.0.0	R v4.3.3		Nucleotide homolog for KMA; protein homolog for SARG mapping
Software	FastQC	v0.12.1			
Software	MultiQC	v1.21			
Software	fastp	v0.23.4			
Database	CARD	v3.2.9			
Database	MobileGeneticElementDatabase	2021-08-16			Accessed: 2024-06-25; last updated: 2017-12-28.
Software	KMA	v1.4.9		-1t1 -mem_mode -ex_mode	
Software	Mosdepth	v0.3.6			
Software	SAMtools	v1.19.2			
Database	SARG	v2023-04-21			
Software	blastp				
Metadata	SARG Risk Ranking	v2022-09-27			
Database	PubMLST	Downloaded on 2024-11-27			
Software	CD-HIT	v4.8.1		-c 0.95 -aS 0.95	
Software	nf-core/taxprofiler	v1.1.7		--run_metaphlan	
Database	ChocoPhlAnSGB	vJun23_202307			
Software	MetaPhlAn	v4.1.1			

Software	ANCOM-BC	v1.2.0	R v4.1.3	p_adj_method = "bonferroni", zero_cut = 1, global = TRUE, conserve = TRUE	
Software	microbiome::	v1.26.0	R v4.4.2		
Software	microViz::	v0.12.6	R v4.4.2		
Software	caret::	v7.0-1	R v4.4.2		
Software	ggplot2::	v3.5.2	R v4.4.2		
Software	rstatix::	v0.7.2	R v4.4.2		
Software	ggpubr::	v0.6.0	R v4.4.2		
Software	ggraph::	v2.2.2	R v4.5.0		
Software	igraph::	v2.2.1	R v4.5.0		
Software	vegan::	v2.6-10	R v4.4.2		
Software	ggalign::	v1.0.2	R v4.4.2		
Software	ComplexHeatmap::	v2.20.0	R v4.4.2		
Software	microshades::	v1.13	R v4.4.2		

Table S4: List of antibiotic resistance genes (ARGs) with identical amino acid sequences in CARD (v3.2.9) and SARG (v2023-04-21) databases, along with their assigned SARG risk levels. (**See Excel file**)

Table S5: Concentrations of antibiotics (ng/L) detected in influent and effluent wastewater samples from the three WWTPs (Gavà-Viladecans (G-V), El Prat de Llobregat (P.LL), and Sant Feliu de Llobregat (F.LL)). Data represent mean concentrations ± S.D. across three sampling weeks (n=3). ND; not detected; < LOQ, below limit of quantification.

Sample Type	Antibiotic	P.LL	F.LL	G-V
Influent	Acetyl-sulfamethoxazole	1322 ± 237	323 ± 37	500 ± 61
Effluent		37 ± 9	ND	21 ± 6
Influent	Azithromycin	711 ± 168	1404 ± 471	774 ± 6
Effluent		523 ± 158	494 ± 14	285 ± 49
Influent	Ciprofloxacin	1022 ± 111	632 ± 57	928 ± 111
Effluent		209 ± 34	334 ± 34	288 ± 41
Influent	Clarithromycin	136 ± 37	638 ± 46	113 ± 43
Effluent		88 ± 24	472 ± 174	57 ± 5
Influent	Doxycycline	3168 ± 338	4335 ± 2327	358 ± 32
Effluent		1493 ± 181	2239 ± 1298	358 ± 115
Influent	Enrofloxacin	785 ± 457	ND	ND
Effluent		92 ± 17	ND	ND
Influent	Levofloxacin	297 ± 49	133 ± 22	335 ± 94
Effluent		113 ± 8	110 ± 6	169 ± 26
Influent	Ofloxacin	792 ± 230	513 ± 108	1819 ± 454
Effluent		215 ± 21	271 ± 9	329 ± 22
Influent	Rifaximin	1189 ± 306	1744 ± 468	536 ± 112
Effluent		87 ± 32	ND	ND
Influent	Sulfamethoxazole	630 ± 59	598 ± 41	680 ± 69
Effluent		414 ± 39	290 ± 29	398 ± 80
Influent	Trimethoprim	180 ± 11	230 ± 12	71 ± 14
Effluent		178 ± 22	270 ± 29	33 ± 8

Table S6: Mean concentrations (± S.D.) of antibiotics (ng/g) detected in sludge samples from the three WWTPs across three sampling weeks: (Gavà- Viladecans (G-V), El Prat de Llobregat (P.LL), and Sant Feliu de Llobregat (F.LL)). Data represent mean concentrations ± S.D. across three sampling weeks (n=3); < LOQ, below limit of quantification.

**Sample Type**	**Antibiotics**	**P.LL**	**F.LL**	**G-V**
Sludge	Azithromycin	146 ± 9	89 ± 12	63 ± 5
Sludge	Ciprofloxacin	352 ± 167	789 ± 171	965 ± 80
Sludge	Doxycycline	22 ± 6	27 ± 5	9 ± 2
Sludge	Enrofloxacin	289 ± 77	115 ± 19	21 ± 1
Sludge	Levofloxacin	253 ± 60	361 ± 101	371 ± 45
Sludge	Miconazole	< LOQ	9 ± 1	10 ± 2
Sludge	Norfloxacin	155 ± 12	181 ± 11	144 ± 4
Sludge	Ofloxacin	495 ± 83	512 ± 19	544 ± 25
Sludge	Rifaximin	174 ± 17	42 ± 3	29 ± 1

Text S1. The following ribosomal subunit essential single-copy marker genes (ESCMG) in prokaryotes were used to estimate the number of prokaryotic cells in sample metagenomes, from Nayfach and Pollard (2015): *rplA*, *rplB*, *rplC*, *rplD*, *rplE*, *rplF*, *rplJ*, *rplK*, *rplM*, *rplN*, *rplO*, *rplP*, *rplR*, *rplV*, *rplX*, *rpsB*, *rpsC*, *rpsE*, *rpsG*, *rpsH*, *rpsI*, *rpsK*, *rpsL*, *rpsM*, *rpsO*, *rpsQ*, *rpsS*.
